# Contributions of the life expectancy gap reduction between urban and rural areas to the increase in overall life expectancy in South Korea from 2000 to 2019

**DOI:** 10.1186/s12939-023-01960-0

**Published:** 2023-07-28

**Authors:** Ikhan Kim

**Affiliations:** Department of Medical Humanities and Social Medicine, Kosin University College of Medicine, 262 Gamcheon-ro, Seo-gu, Busan, Republic of Korea

**Keywords:** Age factors, Chronic disease, Life Expectancy, Rural Population

## Abstract

**Background:**

This study aimed to quantify the contribution of narrowing the life expectancy gap between urban and rural areas to the overall life expectancy at birth in Korea and examine the age and death cause-specific contribution to changes in the life expectancy gap between urban and rural areas.

**Methods:**

We used the registration population and death statistics from Statistics Korea from 2000 to 2019. Assuming two hypothetical scenarios, namely, the same age-specific mortality change rate in urban and rural areas and a 20% faster decline than the observed decline rate in rural areas, we compared the increase in life expectancy with the actual increase. Changes in the life expectancy gap between urban and rural areas were decomposed into age- and cause-specific contributions.

**Results:**

Rural disadvantages of life expectancy were evident. However, life expectancies in rural areas increased more rapidly than in urban areas. Life expectancy would have increased 0.3–0.5 less if the decline rate of age-specific mortality in small-to-middle urban and rural areas were the same as that of large urban areas. Life expectancy would have increased 0.7–0.9 years further if the decline rate of age-specific mortality in small-to-middle urban and rural areas had been 20% higher. The age groups 15–39 and 40–64, and chronic diseases, such as neoplasms and diseases of the digestive system, and external causes significantly contributed to narrowing the life expectancy gap between urban and rural areas.

**Conclusion:**

Pro-health equity interventions would be a good strategy to reduce the life expectancy gap and increase overall life expectancy, particularly in societies where life expectancies have already increased.

**Supplementary Information:**

The online version contains supplementary material available at 10.1186/s12939-023-01960-0.

## Background

Life expectancy at birth (LE) has consistently increased in South Korea (hereafter, Korea). Men’s LE has increased from 72.3 years in 2000 to 76.8 years in 2010 and 80.3 years in 2019, an increase of 4.5 and 3.5 years every decennial period. In the same period, women’s LE increased by 3.9 and 2.7 years (79.7 years in 2000, 83.6 years in 2010, and 86.3 years in 2019). This pattern differs from other countries, especially during the most recent decennial period [1]. For instance, during the first six years of the second decennial period in the United Kingdom, LE in men increased by roughly 0.8 years, from 78.6 (in 2010) to 79.4 years (in 2016). LE in women increased from 82.6 to 83.0 years during the same period [1]. LE in the United States stagnated during the 2010s [2].

One of Korea’s main health policy goals is eliminating regional health gaps. Improving overall health and achieving health equity between income levels and regions are the two overarching goals of the most recent Health Plan 2030 [3]. Since the 1990s, the mortality gap between urban and rural areas in Korea has continuously decreased. The mortality rate in urban areas was consistently lower than that in rural areas, according to a previous study that examined mortality trends in Korea for 20 years, starting in 1993 [4]. However, the mortality rate decreased more rapidly in rural areas than in urban areas, and the mortality gap between the two areas decreased. A more rapid reduction in avoidable mortality in rural areas, such as with cancer and cardiovascular diseases, has narrowed the mortality gap [5]. This situation in Korea offers a good opportunity to examine whether closing the life expectancy gap between urban and rural areas has increased overall life expectancy.

A previous study conducted in Korea found that inequality in self-rated health within a district increases with poorer self-rated health [6]. Recent research has argued that reducing inequality is critical for improving overall health, and understanding the epidemiology by age and cause of death is required to reduce health inequality [7]. However, Anand claimed that achieving health equity and enhancing overall health could constitute normative trade-offs [8]. Despite the theoretical and political justifications, few studies have empirically examined the effect of reducing health inequality at the overall health level.

This study aims to quantify the contribution of narrowing the LE gap between urban and rural areas in Korea to the country’s overall increase in LE. The differences between simulated and actual LEs were estimated when the rate of decrease in mortality in rural areas was equal to that in urban areas and when the decrease in mortality in rural areas was 20% greater than the actual rate. Decomposition analysis was also performed to examine age- and cause-specific contributions to reducing the urban-rural LE gap.

## Methods

### Data

This study used the 2000–2019 whole population and death statistics of Korea provided by Korean Statistics. Korean Statistics publicly releases population data based on resident registration and vital statistics through the Korean Statistical Information Service. Annual population data were obtained by calculating the average population in January and December of each year. Annual population statistics by sex, 5-year age group (0,1,5–9…100+), and district were acquired. In Korea, it is mandatory by law to file a death report with a doctor’s death certificate attached within one month of knowing about the death. The cause of death was included in the death reports, along with information such as sex, age, and residence. The Korean Standard Classification of Diseases and Causes of Death, Seventh Revision, based on the International Classification of Diseases, Tenth Revision, was used [9]. Using the MicroData Integrated Service of Statistics Korea, we obtained annual death data by gender/5-year age group/district/cause of death. Similar to previous studies, we reclassified the causes of death into 15 major categories based on the World Health Organization classification [10, 11]. Supplementary Table S1 lists the detailed classifications of the causes of death.

### Urban-rural definition

The urban-rural definition varies depending on the country and context, and no single representative definition exists [12]. A definition using the district of the administrative unit has the advantage of being the most used, easy to understand, and effective for comparisons of the results [12]. Korea’s districts have the lowest level of autonomous local governments and are classified into three categories: *Gu, Si*, and *Gun* (ordered by urbanity), mainly according to the population size. This study uses the *Gu*, *Si*, and *Gun* classification to define urban and rural areas in Korea. As of 2019, 114 *Gu*s were classified as large urban areas (LUAs), 80 *Si*s as small-to-middle urban areas (SMUAs), and 82 *Gun*s as rural areas (RAs). Population densities according to LUAs, SMUAs, and RAs are shown in Supplementary Table S2.

### Statistical analysis

Considering the stagnation of the LE increase in several countries since 2010, we separated the entire study period into two segments: 2000–2009 and 2010–2019. This study used 100 years or older as the open age interval to calculate the LE [13]. In all analyses, using 2,000 randomly drawn samples by the Monte Carlo simulation approach, a 95% uncertainty interval (UI) was produced, with 2.5 and 97.5 percentiles as the boundary. The number of deaths by sex, year, area (LUAs, SMUAs, and RAs), age group, and cause of death was assumed to follow a Poisson distribution in the Monte Carlo simulation method. All the analyses were stratified according to sex.

We conducted a nonparametric simulation analysis. Two scenarios were proposed. In the first scenario, the difference between the simulated LE increase when age-specific mortality in RAs decreased was considered at the same rate as in LUAs, and the actual overall LE increase was estimated. In the second scenario, we estimated the difference between the simulated LE increase when the age-specific mortality decrease in RAs was 20% faster than the observed rate and the actual overall LE increase. A standard of 20% was chosen based on the relative difference between age-standardized all-cause mortality in patients with RAs and LUAs in 2019. The results in which the mortality rates in RAs and SMUAs declined at the same rate are also presented.

Finally, the LE gap between the LUAs and RAs was decomposed by age group and cause of death. The contour decomposition method was applied [14], an approach that considers the non-additive nature of the LE estimator. The difference between the two areas of the starting- and ending-point LEs and the difference in LE changes between the two areas were decomposed. In the sensitivity analysis, we decomposed the difference between SMUAs and RAs. In addition, considering the variability of urban-rural definitions, another decomposition analysis was performed by reclassifying LUAs, SMUAs, and RAs according to population density. Approximately 90% of the urbanization level was posited as the population density cut-off for categorizing regions into LUAs and SMUAs (corresponding to the top 60th percentile population density districts). Districts with populations in the top 30th percentile of population density were categorized as LUAs.

## Results

**Fig. 1:**
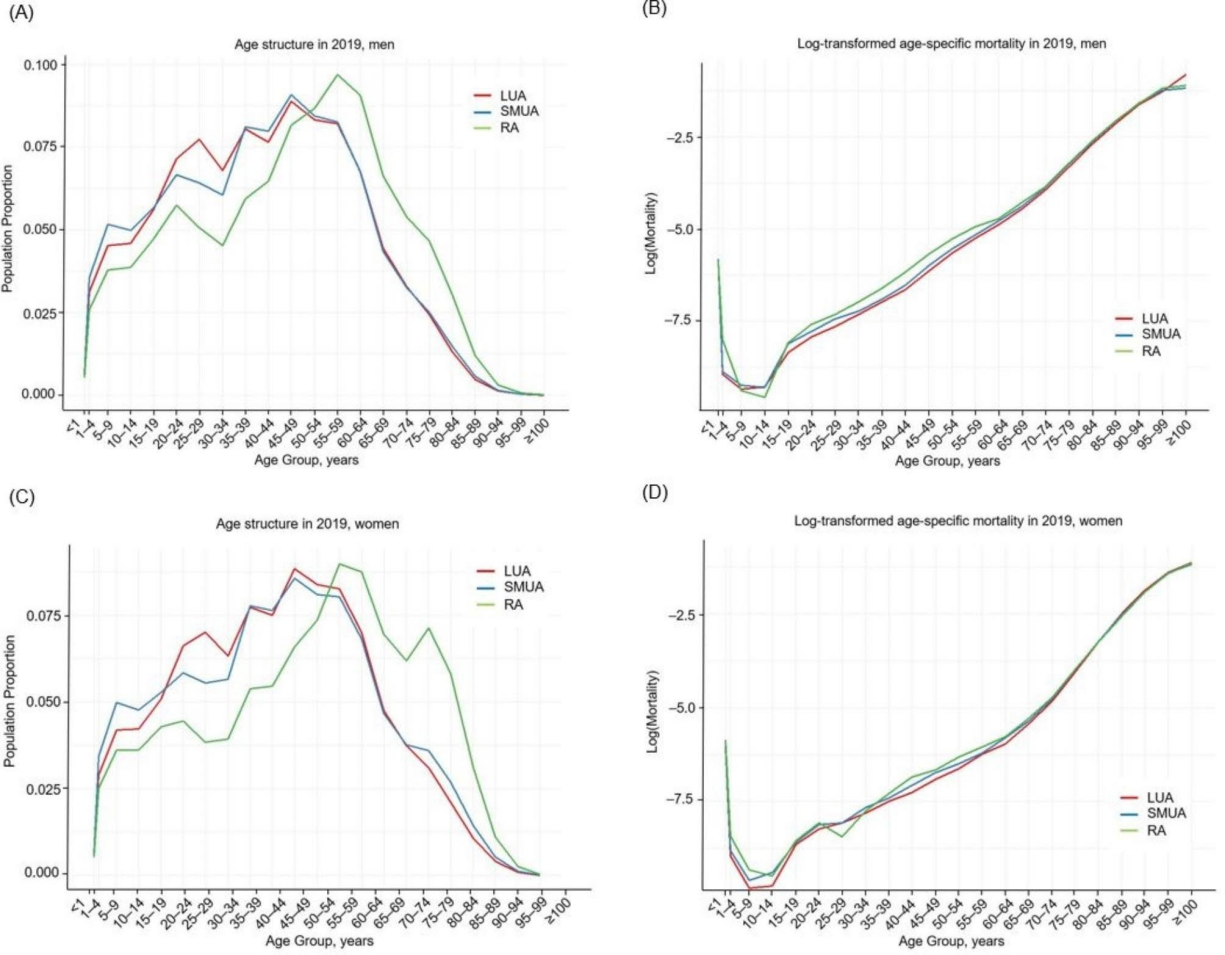
Age structure and log-transformed age-specific mortality by degree of urbanization in 2019: Findings from the Korean Statistical Information Services, Men and women (**A**) Age structure by degree of urbanization in 2019, men (**B**) Log-transformed age-specific mortality by degree of urbanization in 2019, men (**C**) Age structure by degree of urbanization in 2019, women (**D**) Log-transformed age-specific mortality by degree of urbanization in 2019, women LUA = Large Urban Areas; SMUA = Small-to-Middle Urban Areas; RA = Rural Areas

The age distribution and log-transformed all-cause age-specific mortality rates for LUAs, SMUAs, and RAs in 2019 are shown in Fig. 1. Age distribution was inversely U-shaped in all areas. Compared with RAs, the age structure in urban areas was substantially lower for both sexes. In all three areas, the log-transformed age-specific mortality exhibited a J-shape. However, those in RAs had higher mortality rates in the young and middle-aged groups than those in LUAs and SMUAs. Additionally, the mortality gap was wider among men than among women. The age-specific population and mortality figures for all three areas in 2000, 2010, and 2019 are presented in Supplementary Tables S3 and S4, respectively. Approximately 60%, 32%, and 8% of the population belonged to LUAs, SMUAs, and RAs, respectively. However, LUAs, SMUAs, and RAs accounted for approximately 52%, 32%, and 16% of all fatalities, respectively.

Supplementary Fig. 1 shows the sex-specific annual LE trends for the three areas throughout the study. LE was higher in women than men throughout the study period and across all areas. According to urbanicity, in both sexes, LE was the highest in the order of LUAs, SMUAs, and RAs. The LE showed an upward trend, although the rate of increase was slower from 2010 to 2019 than from 2000 to 2009. Compared with LUAs and SMUAs, the LE increase rate in RAs was higher. Thus, the gap in LE between urban and rural areas narrowed. The gap was particularly narrow among women in the 2010s, who initially showed a lower LE gap between urban and rural areas than among men.

The total absolute and relative increases in LE are shown in Table 1, along with comparisons of the increases in the three areas by decade. In terms of absolute change, the total LE for men increased by 4.5 years (95% UI: 4.5, 4.6 years) and by 6.3% (95% UI: 6.2, 6.4%) between 2000 and 2009, and by 3.5 years (95% UI: 3.4, 3.5 years) and 4.5% (95% UI: 4.4, 4.6%) between 2010 and 2019. In all periods, absolute and relative increases rose from low to high urbanicity. Women showed similar trends to men. However, women’s absolute and relative increases were lower than men’s.

**Table 1:** Absolute and relative increases in life expectancy by total and degree of urbanization for the 2000–2009 and 2010–2019 periods: Findings from the Korean Statistical Information Services, 2000–2019, Men and women

	2000–2009				2010–2019			
	Year 2000, yrs (A)	Year 2009, yrs (B)	Absolute increase, yrs (B)-(A)	Relative increase, % (B)-(A) / (A)	Year 2010, yrs (C)	Year 2019, yrs (D)	Absolute increase, yrs (D)-(C)	Relative increase, % (D)-(C) / (C)
**Men**								
Total	72.3 (72.2, 72.4)	76.8 (76.8, 76.9)	4.5 (4.5, 4.6)	6.3 (6.2, 6.4)	77.0 (76.9, 77.0)	80.4 (80.4, 80.5)	3.5 (3.4, 3.5)	4.5 (4.4, 4.6)
LUAs	73.5 (73.4,73.5)	77.5 (77.4,77.6)	4.0 (3.9,4.2)	5.5 (5.4,5.7)	77.6 (77.5,77.7)	80.9 (80.8,80.9)	3.3 (3.1,3.4)	4.2 (4.1,4.4)
SMUAs	71.3 (71.1,71.4)	76.3 (76.2,76.5)	5.1 (4.9,5.2)	7.1 (6.9,7.4)	76.4 (76.3,76.6)	80.1 (80.0,80.2)	3.7 (3.5,3.8)	4.8 (4.6,5.0)
RAs	69.1 (68.9,69.3)	74.6 (74.4,74.8)	5.6 (5.3,5.8)	8.0 (7.6,8.5)	74.9 (74.7,75.1)	79.0 (78.8,79.2)	4.1 (3.8,4.3)	5.4 (5.1,5.8)
**Women**								
Total	79.6 (76.6, 79.7)	83.6 (83.5, 83.7)	4.0 (3.9, 4.1)	5.0 (4.9, 5.1)	83.8 (83.8, 83.9)	86.4 (86.4, 86.5)	2.6 (2.5, 2.7)	3.1 (3.0, 3.2)
LUAs	79.9 (79.8,80.0)	83.8 (83.7,83.9)	3.8 (3.7,4.0)	4.8 (4.6,5.0)	83.9 (83.8,84.0)	86.6 (86.5,86.6)	2.7 (2.5,2.8)	3.2 (3.0,3.3)
SMUAs	86.6 (86.5,86.6)	83.4 (83.3,83.5)	4.1 (4.0,4.3)	5.2 (5.0,5.4)	83.7 (83.6,83.8)	86.3 (86.2,86.4)	2.6 (2.4,2.7)	3.1 (2.9,3.3)
RAs	78.5 (78.4,78.7)	82.9 (82.7,83.1)	4.4 (4.1,4.7)	5.6 (5.3,5.9)	83.3 (83.1,83.5)	86.0 (85.9,86.2)	2.7 (2.5,3.0)	3.3 (3.0,3.6)

LUAs = Large Urban Areas; SMUAs = Small-to-Middle Urban Areas; RAs = Rural Areas

The numbers in parentheses indicate the boundary values of the 95% uncertainty interval

The results from the hypothetical scenario, in which the age-specific mortality decrease rate of RAs was the same as that of LUAs (Scenario 1) and 20% faster than the observed RAs decrease rate (Scenario 2), are shown in Table 2. In Scenario 1, men’s overall LE increased by 0.2 years less than the actual increase (95% UI: -0.2, -0.1 years), whereas in Scenario 2, men’s LE increased by 0.3 years more than the actual increase (95% UI: 0.2, 0.4) in 2000–2009. In women, narrowing the gap in age-specific mortality rates between urban and rural areas also led to larger LE increases during both periods, but the magnitudes were relatively small compared to men. When SMUAs and RAs simultaneously had steeper age-specific mortality decrease rates, the magnitudes of the LE increases were much larger (see Table 3). Relative hypothetical LE increase or decrease in 2010–2019 was greater than in 2000–2009 in all scenarios.

**Table 2:** Absolute increase and difference from the observed increase in overall life expectancy in two hypothetical scenarios for the 2000–2009 and 2010–2019 periods: Findings from the Korean Statistical Information Services, 2000–2019, Men and women

	2000–2009				2010–2019			
	Year 2000, yrs (A)	Year 2009, yrs (B)	Absolute increase, yrs (B)-(A)	Difference from the observed increase, yrs	Year 2010, yrs (C)	Year 2019, yrs (D)	Absolute increase, yrs (D)-(C)	Difference from the observed increase, yrs
**Men**								
Observed	72.3 (72.2, 72.4)	76.8 (76.8, 76.9)	4.5 (4.5, 4.6)		77.0 (76.9, 77.0)	80.4 (80.4, 80.5)	3.5 (3.4, 3.5)	
Scenario 1		76.7 (76.6, 77.8)	4.4 (4.3, 4.5)	-0.2 (-0.2, -0.1)		80.3 (80.2, 80.4)	3.3 (3.2, 3.4)	-0.1 (-0.2, 0.0)
Scenario 2		77.2 (77.1, 77.2)	4.9 (4.8, 4.9)	0.3 (0.2, 0.4)		80.7 (80.6, 80.7)	3.7 (3.6, 3.8)	0.3 (0.2, 0.3)
**Women**								
Observed	79.6 (76.6, 79.7)	83.6 (83.5, 83.7)	4.0 (3.9, 4.1)		83.8 (83.8, 83.9)	86.4 (86.4, 86.5)	2.6 (2.5, 2.7)	
Scenario 1		83.5 (83.4, 83.5)	3.8 (3.7, 3.9)	-0.1 (-0.2, -0.1)		86.3 (86.3, 86.4)	2.5 (2.4, 2.6)	-0.1 (-0.2, 0.0)
Scenario 2		83.9 (83.8, 83.9)	4.3 (4.2, 4.3)	0.3 (0.2, 0.4)		86.7 (86.6, 86.7)	2.8 (2.8, 2.9)	0.2 (0.1, 0.3)

In Scenario 1, age-specific mortality in rural areas (RAs) was hypothesized to decrease at the same rate as in large urban areas (LUAs). In Scenario 2, the age-specific mortality decrease in RA was hypothesized to be 20% faster than the observed rate

The numbers in parentheses indicate the boundary values of the 95% uncertainty interval

**Table 3:** Absolute increase and difference from the observed increase in overall life expectancy in two hypothetical scenarios when the same mortality change rates in small-to-middle urban areas (SMUAs) and rural areas (RAs) were posited for the 2000–2009 and 2010–2019 periods: Findings from the Korean Statistical Information Services, 2000–2019, Men and women

	2000–2009				2010–2019			
	Year 2000, yrs (A)	Year 2009, yrs (B)	Absolute increase, yrs (B)-(A)	Difference from the observed increase, yrs	Year 2010, yrs (C)	Year 2019, yrs (D)	Absolute increase, yrs (D)-(C)	Difference from the observed increase, yrs
**Men**								
Observed	72.3 (72.2, 72.4)	76.8 (76.8, 76.9)	4.5 (4.5, 4.6)		77.0 (76.9, 77.0)	80.4 (80.4, 80.5)	3.5 (3.4, 3.5)	
Scenario 1		76.3 (76.2, 76.4)	4.0 (3.9, 4.1)	-0.5 (-0.6, -0.4)		80.0 (80.0, 80.1)	3.1 (3.0, 3.2)	-0.4 (-0.5, -0.3)
Scenario 2		77.7 (77.6, 77.8)	5.4 (5.3, 5.5)	0.9 (0.8, 0.9)		81.2 (81.2, 81.3)	4.2 (4.2, 4.3)	0.8 (0.7, 0.9)
**Women**								
Observed	79.6 (76.6, 79.7)	83.6 (83.5, 83.7)	4.0 (3.9, 4.1)		83.8 (83.8, 83.9)	86.4 (86.4, 86.5)	2.6 (2.5, 2.7)	
Scenario 1		83.2 (83.2, 83.3)	3.6 (3.5, 3.7)	-0.4 (-0.5, -0.3)		86.2 (86.1, 86.2)	2.4 (2.3, 2.5)	-0.3 (-0.4, -0.2)
Scenario 2		84.4 (84.3, 84.5)	4.8 (4.7, 4.9)	0.8 (0.7, 0.9)		87.1 (87.0, 87.2)	3.3 (3.2, 3.4)	0.7 (0.6, 0.7)

In Scenario 1, age-specific mortalities in the SMUA and RA groups were hypothesized to decrease at the same rate as in the LUA group. In Scenario 2, the age-specific mortality decrease in SMUAs and RAs was hypothesized to be 20% faster than the observed rate

The numbers in parentheses indicate the boundary values of the 95% uncertainty interval

**Fig. 2:**
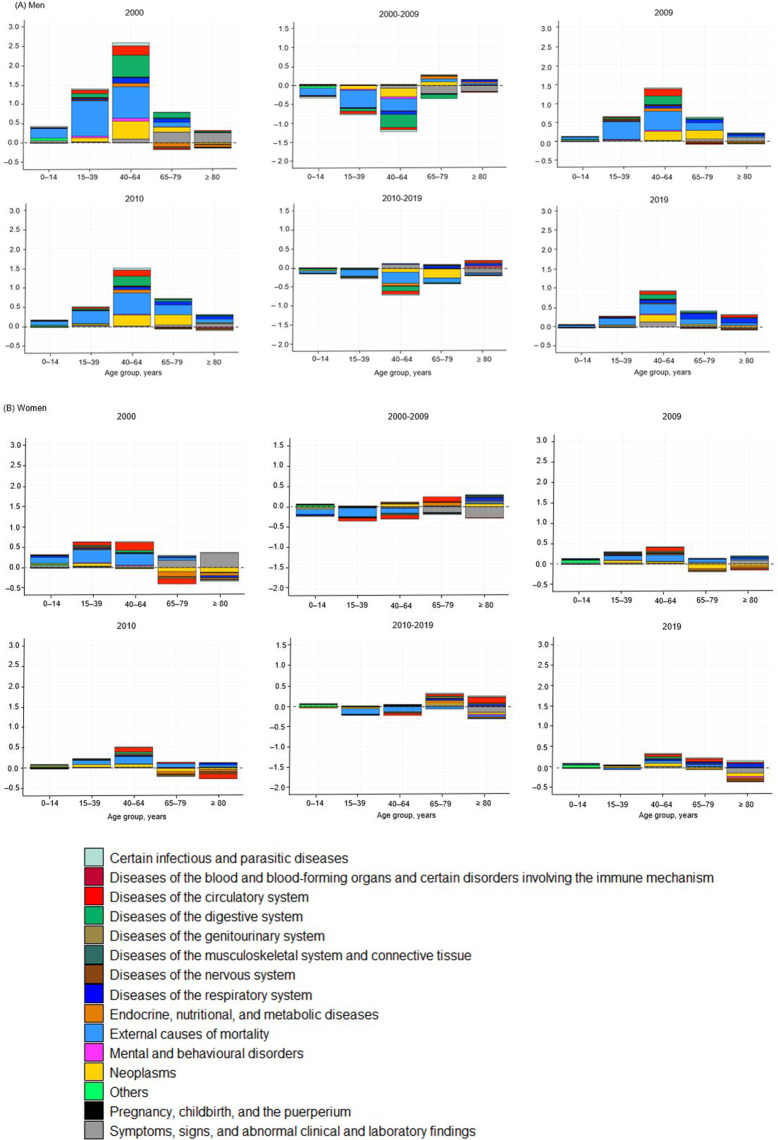
Age- and cause-specific contribution to the life expectancy (LE) gap between large urban areas (LUAs) and rural areas (RAs) in 2000−2009 and 2010−2019: Findings from the Korean Statistical Information Services, Men and women A positive direction indicates a higher LE in the LUAs and a negative direction indicates a higher LE in the RAs. The middle two panels represent the differences in LE changes between 2000−2009 and 2010−2019. The positive direction also indicates higher LE increases in the LUAs, and the negative direction indicates higher LE increases in the RAs.

Figure 2 shows the results of the contour decomposition analysis of the LUAs and RAs. A positive direction indicated higher values in the LUAs, whereas a negative direction indicated higher values in the RAs. The ages 15–39 and 40–64 years were the main contributors to the LE gap between LUAs and RAs in men. Regarding the cause of death, external causes, diseases of the digestive and circulatory systems, and neoplasms contributed more than other causes (in 2000 and 2010). However, the mortality gap between LUAs and RAs in the young and middle-aged groups was narrow. Among the causes of death, external causes, digestive system diseases, and neoplasms have largely contributed to the reduction in mortality. Women’s LE gaps between LUAs and RAs were mainly noted at the ages of 15–39 and 40–64 years, and external causes and diseases of the circulatory system largely contributed to this gap. Women, similar to men, showed a narrowing of the LE gap, which young and middle-aged groups and external causes of death mostly drove. Supplementary Tables S5–S7 present the point estimates and 95% UI of the contour decomposition analysis.

Supplementary Figures S2 and S3 show the results of the sensitivity analysis. A pattern similar to that of the LE gap between the LUAs and RAs was observed when the gaps between the SMUAs and RAs were decomposed. However, each contribution was smaller because the magnitudes of the overall LE gaps between SMUAs and RAs were smaller than those between LUAs and RAs. The results of the decomposition analyses utilizing population density as the criterion for the new definitions of LUAs, SMUAs, and RAs are shown in Supplementary Figure S2. As seen in the primary findings, in the young and middle-aged groups, external causes, diseases of the digestive and circulatory systems, and neoplasms in men, and external causes and diseases of the circulatory systems in women accounted for the LE gaps between LUAs and RAs. The LE gap between LUAs and RAs decreased because of mortality.

## Discussion

This study showed that LE in Korea increased in both men and women from 2000 to 2019, but the rate of increase decreased. In particular, the faster LE increase in the RA group contributed to a greater increase in overall LE in both sexes. The LE gap between urban and rural areas appeared mainly in young and middle-aged groups. External causes, circulatory and digestive system diseases, and neoplasms contributed significantly. However, these age groups and the causes of death contributed to a reduction in the LE gap throughout the study period.

Since the 2000s, LE in Korea has steadily increased. A previous study found that since the 1970s, LE in Korea has increased steadily and rapidly, exceeding the average increase rate of LE in member countries of the Organization for Economic Co-operation and Development (OECD) [15]. Previous research examining the increase in LE between 1998 and 2017 revealed that a major driver of the increase in LE was the decline in avoidable mortality from diseases such as cardiovascular diseases, neoplasms, and transport injuries [16]. The decrease in avoidable mortality was faster than that of all-cause mortality in Korea, and the rate of decrease was more prominent in RAs than in LUAs or SMUAs [4, 5].

This study found that faster increases in RAs and SMUAs enhanced the overall increase in LE in Korea than in LUAs. The LE estimator uses the age-specific probabilities of death and survival as factors [13]. Because of the non-additive nature of the LE estimator, previous research has shown that as LE increases, a greater mortality reduction is necessary further to increase the LE by one unit [17]. Thus, the increase in LE slows down or stagnates in countries where LE has already increased [17]. Therefore, reducing mortality rates in subgroups with relatively high mortality rates (in our study, people in less urbanized areas) would alleviate the stagnation of LE increases in many countries where LE has already increased. The large relative increase in the second scenario in 2010–2019, when the LE was higher than in 2000−2009, can confirm this argument. Furthermore, the effect was more pronounced when the proportion of the affected population was high.

There is an LE gap between urban and rural areas in Korea for both men and women. Previous studies identified compositional and contextual effects at the population level as the causes of variations in mortality between regions [18]. In particular, it is generally known that rural areas in many countries have a lower socioeconomic status than urban areas and are in more poverty, resulting in a mortality gap between urban and rural areas [19]. The rural mortality penalty has also been attributed to several factors, including poor access to healthcare, income inequality, poorer nutrition, unhealthy lifestyle choices, and residential selection in rural areas [19, 20]. Rural residents in Korea are more likely than urban residents to have a lower socioeconomic status and endure multifaceted poverty [21]. In addition, rural areas have a high proportion of older people aged over 80 years (see Fig. 1) and low rates of healthy behaviors [22].

The LE gap between urban and rural areas in Korea decreased for both men and women during the study period. External causes (in both sexes), neoplasms, digestive system diseases (in men), and circulatory system diseases (in women) were the key factors contributing to the narrowing of the LE gap. Among external causes and diseases of the digestive system, traffic accidents, intentional self-harm, and liver diseases showed a considerable decline in mortality (data available on request). According to a previous study, rural areas showed a higher absolute mortality reduction than urban areas, although their relative sizes were similar (rural areas: 54.6%, urban areas: 56.8%). The avoidable mortality rate decreased more rapidly, by 63.5% in urban areas and 61.6% in rural areas. The expansion of a centralized healthcare policy, improvement in material living standards in rural areas, and a special budget for areas with poor health and medical facilities were all nominated as causes of a similar relative decrease in mortality in urban and rural areas [5]. Further research is required to determine which policy factors or processes causally contribute to this decrease.

External causes of mortality (traffic accidents and intentional self-harm) and liver diseases, among the factors contributing to the reduction in mortality, are often socially determined and hence called social pathologies [23]. In Korea, a national traffic accident control policy was established in 1999, and extensive measures such as seatbelt use, speed limits, a campaign on drunk driving, and increased pedestrian safety were implemented nationally [24]. Korea’s suicide rate was among the highest among OECD countries and grew steadily from the 1990s to the early 2010s [25]. In 2004, the government established a central strategy for addressing suicide, updated every five years [26]. The regulation of paraquat effectively prevented suicides in rural areas, where the suicide incidence related to pesticide use was higher than that in urban areas [27]. Further, implementing the hepatitis preventive vaccine program was credited with declining liver disease-related mortality in Korea [28].

This study had some limitations. First, the population movements were not captured. It is well known that the population movement between urban and rural areas is active and that various life events are associated [29]. Given the situation in Korea, where there is an increasing issue of medical accessibility and various infrastructure shortages due to the diminishing rural population, it is possible that the reduction in the life expectancy gap between urban and rural areas is influenced by older people from rural areas relocating to urban areas in search of care from their families or medical facilities [30]. Therefore, the findings of this study should be interpreted as an outcome of estimating the health level of the regional population at that time rather than regarded as having aerial effects on health. Second, rather than using a single representative method, the definition of urban and rural areas varies by country and study purpose [12]. In a Korean study, the degree of urbanization was classified using *Dong, Eup*, and *Myeon*, one level lower local administrative unit than that of *Gu, Si*, and *Gun* [31]. However, districts (*Gu, Si*, and *Gun)* are the smallest politically independent units, relatively stable, and easy to compare with other research results. A similar pattern was observed when the districts were reclassified using population density in the sensitivity analysis. Third, even if every cause of death in Korea is documented under a physician’s diagnosis, it is possible that the cause of death was incorrectly or differently categorized depending on the period.

## Conclusions

Narrowing the LE gap between urban and rural areas has contributed to an overall increase in LE in Korea. Decreases in premature and avoidable mortality, including mortality in the young and middle-aged groups and mortality from external causes, neoplasms, and diseases of the digestive and circulatory systems, mostly contributed to the narrowing of the LE gap between urban and rural areas. Efforts to reduce health inequalities would also improve society’s overall health, particularly when the health level has increased. It is necessary to identify the causes and pathways that contribute to the reduction of mortality in each area, as well as to pursue effective policy measures aimed at further narrowing the life expectancy gap between urban and rural areas.

## Electronic supplementary material

Below is the link to the electronic supplementary material.

**Supplementary Table S1**. List of categories and corresponding ICD-10 codes of the WHO International Statistical Classification of Diseases and Related Health Problems, 10th revision (ICD-10) mortality tabulation list. **Supplementary Table S2**. Distribution of population density according to urban and rural definitions in 2007 and 2017: Data from the Korean Statistical Information Services. **Supplementary Table S3**. Population and deaths by age group according to urban and rural classifications in 2000, 2010, and 2019: Data from the Korean Statistical Information Services, Men. **Supplementary Table S4**. Population and deaths by age group according to urban and rural classification in 2000, 2010, and 2019: Data from the Korean Statistical Information Services, Women. **Supplementary Table S5**. Point estimate and 95% uncertainty interval of the contour decomposition analysis at the starting point of each decennial period: Findings from the Korean Statistical Information Services, Men and women. **Supplementary Table S6**. Point estimate and 95% uncertainty interval of the contour decomposition analysis of changes in life expectancy at birth for each period: Findings from the Korean Statistical Information Services, Men and women. **Supplementary Table S7**. Point estimate and 95% uncertainty interval of contour decomposition analysis at the endpoint of each decennial period: Findings from the Korean Statistical Information Services, Men, and women. **Supplementary Figure S1**. Annual trends of life expectancy by degree of urbanization in Korea, 2000–2019: Findings from the Korean Statistical Information Service, Men and women (A) Annual trends of life expectancy by degree of urbanization, men (B) Annual trends of life expectancy by degree of urbanization, women. **Supplementary Figure S2**. Age- and cause-specific contribution to the life expectancy (LE) gap between small-to-middle urban areas (SMUAs) and rural areas (RAs) in 2000–2009 and 2010–2019: Findings from the Korean Statistical Information Services, Men and women. **Supplementary Figure S3**. Age- and cause-specific contribution to the life expectancy (LE) gap between large urban areas (LUAs) and rural areas (RAs) reclassified according to population density in 2000–2009 and 2010–2019: Findings from the Korean Statistical Information Services, Men and women.

## Data Availability

The datasets analyzed in this study are publicly available on the National Statistical Portal of the Korean Statistical Information Service (https://kosis.kr/index/index.do).

## List of abbreviations

LE: Life expectancy at birth
LUA: Large Urban Area
SMUA: Small-to-Middle Urban Area
RA: Rural Area
UI: Uncertainty interval
OECD: Organization for Economic Co-operation and Development
