# Supplementary material for: Contributions of the life expectancy gap reduction between urban and rural areas to the increase in overall life expectancy in South Korea from 2000 to 2019
Source: Int J Equity Health. 2023 Jul 28;22:141. doi: 10.1186/s12939-023-01960-0 (PMC10375755; doi:10.1186/s12939-023-01960-0)
Supplement: Supplementary file 1 — Supplementary Table S1. List of categories and corresponding ICD-10 codes of the WHO International Statistical Classification of Diseases and Related Health Problems, 10th revision (ICD-10) mortality tabulation list. Supplementary Table S2. Distribution of population density according to urban and rural definitions in 2007 and 2017: Data from the Korean Statistical Information Services. Supplementary Table S3. Population and deaths by age group according to urban and rural classifications in 2000, 2010, and 2019: Data from the Korean Statistical Information Services, Men. Supplementary Table S4. Population and deaths by age group according to urban and rural classification in 2000, 2010, and 2019: Data from the Korean Statistical Information Services, Women. Supplementary Table S5. Point estimate and 95% uncertainty interval of the contour decomposition analysis at the starting point of each decennial period: Findings from the Korean Statistical Information Services, Men and women. Supplementary Table S6. Point estimate and 95% uncertainty interval of the contour decomposition analysis of changes in life expectancy at birth for each period: Findings from the Korean Statistical Information Services, Men and women. Supplementary Table S7. Point estimate and 95% uncertainty interval of contour decomposition analysis at the endpoint of each decennial period: Findings from the Korean Statistical Information Services, Men, and women. Supplementary Figure S1. Annual trends of life expectancy by degree of urbanization in Korea, 2000–2019: Findings from the Korean Statistical Information Service, Men and women (A) Annual trends of life expectancy by degree of urbanization, men (B) Annual trends of life expectancy by degree of urbanization, women. Supplementary Figure S2. Age- and cause-specific contribution to the life expectancy (LE) gap between small-to-middle urban areas (SMUAs) and rural areas (RAs) in 2000–2009 and 2010–2019: Findings from the Korean Statistical Informa [file 12939_2023_1960_MOESM1_ESM.docx]

**Table of contents**

Supplementary Table S1. List of categories and corresponding ICD-10 codes of the WHO International Statistical Classification of Diseases and Related Health Problems, 10th revision (ICD-10) mortality tabulation list.

Supplementary Table S2. Distribution of population density according to urban and rural definitions in 2007 and 2017: Data from the Korean Statistical Information Services.

Supplementary Table S3. Population and deaths by age group according to urban and rural classifications in 2000, 2010, and 2019: Data from the Korean Statistical Information Services, Men.

Supplementary Table S4. Population and deaths by age group according to urban and rural classification in 2000, 2010, and 2019: Data from the Korean Statistical Information Services, Women.

Supplementary Table S5. Point estimate and 95% uncertainty interval of the contour decomposition analysis at the starting point of each decennial period: Findings from the Korean Statistical Information Services, Men and women.

Supplementary Table S6. Point estimate and 95% uncertainty interval of the contour decomposition analysis of changes in life expectancy at birth for each period: Findings from the Korean Statistical Information Services, Men and women.

Supplementary Table S7. Point estimate and 95% uncertainty interval of contour decomposition analysis at the endpoint of each decennial period: Findings from the Korean Statistical Information Services, Men, and women.

Supplementary Figure S1. Annual trends of life expectancy by degree of urbanization in Korea, 2000−2019: Findings from the Korean Statistical Information Service, Men and women (A) Annual trends of life expectancy by degree of urbanization, men (B) Annual trends of life expectancy by degree of urbanization, women

Supplementary Figure S2. Age- and cause-specific contribution to the life expectancy (LE) gap between small-to-middle urban areas (SMUAs) and rural areas (RAs) in 2000−2009 and 2010−2019: Findings from the Korean Statistical Information Services, Men and women.

Supplementary Figure S3. Age- and cause-specific contribution to the life expectancy (LE) gap between large urban areas (LUAs) and rural areas (RAs) reclassified according to population density in 2000−2009 and 2010−2019: Findings from the Korean Statistical Information Services, Men and women.

Supplementary Table S1. List of categories and corresponding ICD-10 codes of the WHO International Statistical Classification of Diseases and Related Health Problems, 10th revision (ICD-10) mortality tabulation list.

| **Code** | **Disease** | **ICD-10 code** |
| --- | --- | --- |
| **1** | **Certain infectious and parasitic diseases** | **A00-B99** |
| 1-001 | Cholera | A00 |
| 1-002 | Other gastroenteritis and colitis of infectious and unspecified origin | A09 |
| 1-003 | Other intestinal infectious diseases | A01-A08 |
| 1-004 | Respiratory tuberculosis | A15-A16 |
| 1-005 | Other tuberculosis | A17-A19 |
| 1-006 | Plague | A20 |
| 1-007 | Tetanus | A33-A35 |
| 1-008 | Diphtheria | A36 |
| 1-009 | Whooping cough | A37 |
| 1-010 | Meningococcal infection | A39 |
| 1-011 | Septicaemia | A40-A41 |
| 1-012 | Infections with a predominantly sexual mode of transmission | A50-A64 |
| 1-013 | Acute poliomyelitis | A80 |
| 1-014 | Rabies | A82 |
| 1-015 | Yellow fever | A95 |
| 1-016 | Other arthropod-borne viral fevers and viral hemorrhagic fevers | A90-A94, A96-A99 |
| 1-017 | Measles | B05 |
| 1-018 | Viral hepatitis | B15-B19 |
| 1-019 | Human immunodeficiency virus[HIV] disease | B20-B24 |
| 1-020 | Malaria | B50-B54 |
| 1-021 | Leishmaniasis | B55 |
| 1-022 | Trypanosomiasis | B56-B57 |
| 1-023 | Schistosomiasis | B65 |
| 1-024 | Remainder of certain infectious and parasitic diseases | A21-A32,A38, A42-A49,A65-A79,A81,A83-A89, B00-B04,B06-B09, B25-B49,B58-B64, B66-B94,B99 |
| **2** | **Neoplasms** | **C00-D48** |
| 2-001 | Malignant neoplasms of lip, oral cavity, and pharynx | C00-C14 |
| 2-002 | Malignant neoplasm of esophagus | C15 |
| 2-003 | Malignant neoplasm of stomach | C16 |
| 2-004 | Malignant neoplasms of colon, rectum, and anus | C18-C21 |
| 2-005 | Malignant neoplasms of liver and intrahepatic bile ducts | C22 |
| 2-006 | Malignant neoplasm of pancreas | C25 |
| 2-007 | Malignant neoplasm of larynx | C32 |
| 2-008 | Malignant neoplasms of trachea, bronchus, and lung | C33-C34 |
| 2-009 | Malignant melanoma of skin | C43 |
| 2-010 | Malignant neoplasm of breast | C50 |
| 2-011 | Malignant neoplasm of cervix uteri | C53 |
| 2-012 | Malignant neoplasms of other and unspecified parts of uterus | C54-C55 |
| 2-013 | Malignant neoplasm of ovary | C56 |
| 2-014 | Malignant neoplasm of prostate | C61 |
| 2-015 | Malignant neoplasm of bladder | C67 |
| 2-016 | Malignant neoplasms of meninges, brain, and other parts of central nervous system | C70-C72 |
| 2-017 | Non-Hodgkin's lymphoma | C82-C86 |
| 2-018 | Multiple myeloma and malignant plasma cell neoplasms | C90 |
| 2-019 | Leukaemia | C91-C95 |
| 2-020 | Remainder of malignant neoplasms | C17, C23-C24 C26-C31, C37-C41, C44-C49, C51-C52, C57-C60, C62-C66, C68-C69, C73-C81, C88, C96-C97 |
| 2-021 | Remainder of neoplasms | D00-D48 |
| **3** | **Diseases of the blood and blood-forming organs and certain disorders involving the immune mechanism** | **D50-D89** |
| 3-001 | Anaemias | D50-D64 |
| 3-002 | Remainder of diseases of the blood and blood-forming organs and certain disorders involving the immune mechanism | D65-D89 |
| **4** | **Endocrine, nutritional, and metabolic diseases** | **E00-E88** |
| 4-001 | Diabetes mellitus | E10-E14 |
| 4-002 | Malnutrition | E40-E46 |
| 4-003 | Remainder of endocrine, nutritional, and metabolic diseases | E00-E07, E15-E34, E50-E88 |
| **5** | **Mental and behavioral disorders** | **F01-F99** |
| 5-001 | Mental and behavioral disorders due to psychoactive substance use | F10-F19 |
| 5-002 | Remainder of mental and behavioral disorders | F01-F09, F20-F99 |
| **6** | **Diseases of the nervous system** | **G00-G98** |
| 6-001 | Meningitis | G00,G03 |
| 6-002 | Alzheimer's disease | G30 |
| 6-003 | Remainder of diseases of the nervous system | G04-G25, G31-G98 |
| 6-004 | Diseases of the eye and adnexa | H00-H57 |
| 6-005 | Diseases of the ear and mastoid process | H60-H93 |
| **7** | Diseases of the circulatory system | I00-I99 |
| 7-001 | Acute rheumatic fever and chronic rheumatic heart diseases | I00-I09 |
| 7-002 | Hypertensive diseases | I10-I13 |
| 7-003 | Ischaemic heart diseases | I20-I25 |
| 7-004 | Other heart diseases | I26-I51 |
| 7-005 | Cerebrovascular diseases | I60-I69 |
| 7-006 | Atherosclerosis | I70 |
| 7-007 | Remainder of diseases of the circulatory system | I71-I99 |
| **8** | **Diseases of the respiratory system** | **J00-J98** |
| 8-001 | Influenza | J09-J11 |
| 8-002 | Pneumonia | J12-J18 |
| 8-003 | Other acute lower respiratory infections | J20-J22 |
| 8-004 | Chronic lower respiratory diseases | J40-J47 |
| 8-005 | Remainder of diseases of the respiratory system | J00-J06, |
| **9** | **Diseases of the digestive system** | **K00-K92** |
| 9-001 | Gastric and duodenal ulcer | K25-K27 |
| 9-002 | Diseases of the liver | K70-K76 |
| 9-003 | Remainder of diseases of the digestive system | K00-K22, |
| 9-004 | Diseases of the skin and subcutaneous tissue | L00-L98 |
| **10** | **Diseases of the musculoskeletal system and connective tissue** | **M00-M99** |
| **11** | **Diseases of the genitourinary system** | **N00-N98** |
| 11-001 | Glomerular and renal tubulo-interstitial diseases | N00-N15 |
| 11-002 | Remainder of diseases of the genitourinary system | N17-N98 |
| **12** | **Pregnancy, childbirth, and the puerperium** | **O00-O99** |
| 12-001 | Pregnancy with abortive outcome | O00-O07 |
| 12-002 | Other direct obstetric deaths | O10-O92 |
| 12-003 | Indirect obstetric deaths | O98-O99 |
| 12-004 | Remainder of pregnancy, childbirth, and the puerperium | O95-O97 |
| 12-005 | Certain conditions originating in the perinatal period | P00-P96 |
| 12-006 | Congenital malformations, deformations, and chromosomal abnormalities | Q00-Q99 |
| **13** | **Symptoms, signs, and abnormal clinical and laboratory findings, NEC** | **R00-R99** |
| **14** | **External causes of mortality** | **V01-Y89** |
| 14-001 | Transport accidents | V01-V99 |
| 14-002 | Falls | W00-W19 |
| 14-003 | Accidental drowning and submersion | W65-W74 |
| 14-004 | Exposure to smoke, fire, and flames | X00-X09 |
| 14-005 | Accidental poisoning by and exposure to noxious substances | X40-X49 |
| 14-006 | Intentional self-harm | X60-X84 |
| 14-007 | Assault | X85-Y09 |
| **15** | **All other external causes** | **W20-W64,W75-W99, X10-X39,X50-X59, Y10-Y89** |

Supplementary Table S2. Distribution of population density according to urban and rural definitions in 2007 and 2017: Data from the Korean Statistical Information Services.

|  | **2007** | | | **2017** | |
| --- | --- | --- | --- | --- | --- |
|  | **Median (IQR)** | **(Min, Max)** | **Median (IQR)** | | **(Min, Max)** |
| LUA | 8686.7 (12377.0) | (290.7, 28882.9) | 8326.1 (11247.0) | | (367.2, 26942.5) |
| SMUA | 413.7 (566.4) | (60.1, 8133.6) | 483.9 (773.3) | | (57.6, 15763.7) |
| RA | 76.3 (65.1) | (19.9, 376.6) | 71.7 (70.0) | | (19.7, 729.6) |

LUA, large urban area; SMUA, small-to-middle urban area; RA, rural area; IQR, interquartile range; Min = Minimum; Max = Maximum.

Supplementary Table S3. Population and deaths by age group according to urban and rural classifications in 2000, 2010, and 2019: Data from the Korean Statistical Information Services, Men.

|  | **2000** | | | | | | | | **2010** | | | | | | | **2019** | | | | | | | |
| --- | --- | --- | --- | --- | --- | --- | --- | --- | --- | --- | --- | --- | --- | --- | --- | --- | --- | --- | --- | --- | --- | --- | --- |
|  | **Population** | | | **Deaths** | | | | | **Population** | | | **Deaths** | | | | **Population** | | | | **Deaths** | | | |
|  | **LUA** | **SMUA** | **RA** | | **LUA** | **SMUA** | **RA** | **LUA** | | **SMUA** | **RA** | | **LUA** | **SMUA** | **RA** | | **LUA** | **SMUA** | **RA** | | **LUA** | **SMUA** | **RA** |
| 0 | 197442.5 (0.013) | 90330 (0.014) | 25212.5 (0.011) | | 935 (473.6) | 450 (498.2) | 160 (634.6) | 137635 (0.009) | | 71268 (0.010) | 16227 (0.008) | | 531 (385.8) | 287 (402.7) | 70 (431.4) | | 91469.5 (0.006) | 54088.5 (0.007) | 11657.5 (0.005) | | 271 (296.3) | 161 (297.7) | 33 (283.1) |
| 1-4 | 874107 (0.058) | 408654 (0.063) | 114536 (0.048) | | 371 (42.4) | 251 (61.4) | 86 (75.1) | 577486.5 (0.037) | | 306459 (0.042) | 67354.5 (0.031) | | 116 (20.1) | 72 (23.5) | 25 (37.1) | | 475619.5 (0.031) | 289584 (0.035) | 56792 (0.026) | | 62 (13.0) | 41 (14.2) | 19 (33.5) |
| 5-9 | 1200368.5 (0.080) | 538131.5 (0.083) | 148392 (0.062) | | 305 (25.4) | 195 (36.2) | 85 (57.3) | 813753.5 (0.052) | | 423679 (0.058) | 93854.5 (0.044) | | 99 (12.2) | 55 (13.0) | 21 (22.4) | | 688243.5 (0.045) | 421376 (0.052) | 83394.5 (0.038) | | 60 (8.7) | 41 (9.7) | 7 (8.4) |
| 10-14 | 1073867 (0.072) | 446930 (0.069) | 137260 (0.057) | | 216 (20.1) | 119 (26.6) | 64 (46.6) | 1081390 (0.070) | | 522007 (0.072) | 120039.5 (0.056) | | 132 (12.2) | 92 (17.6) | 30 (25.0) | | 699682.5 (0.046) | 406444.5 (0.050) | 85163.5 (0.039) | | 65 (9.3) | 37 (9.1) | 6 (7.0) |
| 15-19 | 1268386 (0.084) | 513101.5 (0.080) | 192182.5 (0.080) | | 605 (47.7) | 408 (79.5) | 209 (108.8) | 1208139 (0.078) | | 531976 (0.073) | 130755 (0.061) | | 414 (34.3) | 226 (42.5) | 65 (49.7) | | 853320.5 (0.056) | 461539.5 (0.056) | 104078 (0.047) | | 203 (23.8) | 139 (30.1) | 32 (30.7) |
| 20-24 | 1263319.5 (0.084) | 522881.5 (0.081) | 222755 (0.093) | | 924 (73.1) | 582 (111.3) | 299 (134.2) | 1056318 (0.068) | | 454428.5 (0.062) | 128057.5 (0.059) | | 544 (51.5) | 291 (64.0) | 79 (61.7) | | 1088039.5 (0.071) | 544625 (0.067) | 126519.5 (0.057) | | 392 (36.0) | 226 (41.5) | 64 (50.6) |
| 25-29 | 1505963 (0.100) | 588298 (0.091) | 203712 (0.085) | | 1245 (82.7) | 738 (125.4) | 377 (185.1) | 1284933.5 (0.083) | | 531525.5 (0.073) | 135471 (0.063) | | 825 (64.2) | 483 (90.9) | 162 (119.6) | | 1176294 (0.077) | 523427 (0.064) | 111593.5 (0.051) | | 558 (47.4) | 306 (58.5) | 74 (66.3) |
| 30-34 | 1481873.5 (0.099) | 623375.5 (0.097) | 181580 (0.076) | | 1574 (106.2) | 1004 (161.1) | 479 (263.8) | 1287735 (0.083) | | 564189.5 (0.077) | 132641 (0.062) | | 1013 (78.7) | 591 (104.8) | 182 (137.2) | | 1033533.5 (0.068) | 494400.5 (0.061) | 99660 (0.045) | | 684 (66.2) | 355 (71.8) | 93 (93.3) |
| 35-39 | 1447903 (0.096) | 634065 (0.098) | 183260 (0.077) | | 2523 (174.3) | 1533 (241.8) | 758 (413.6) | 1423661.5 (0.092) | | 675886.5 (0.093) | 160660 (0.075) | | 1653 (116.1) | 951 (140.7) | 307 (191.1) | | 1225681 (0.080) | 661955.5 (0.081) | 130725 (0.059) | | 1146 (93.5) | 667 (100.8) | 177 (135.4) |
| 40-44 | 1346722.5 (0.090) | 555942.5 (0.086) | 175155 (0.073) | | 4009 (297.7) | 2334 (419.8) | 1074 (613.2) | 1394938 (0.090) | | 671334.5 (0.092) | 171527.5 (0.080) | | 2730 (195.7) | 1568 (233.6) | 604 (352.1) | | 1165387.5 (0.076) | 651857.5 (0.080) | 142464 (0.065) | | 1510 (129.6) | 948 (145.4) | 297 (208.5) |
| 45-49 | 965716 (0.064) | 370237 (0.057) | 134662.5 (0.056) | | 4589 (475.2) | 2467 (666.3) | 1194 (886.7) | 1372517 (0.088) | | 641965 (0.088) | 181809 (0.084) | | 4432 (322.9) | 2393 (372.8) | 970 (533.5) | | 1352565 (0.089) | 741453.5 (0.091) | 180013 (0.082) | | 2889 (213.6) | 1831 (246.9) | 608 (337.8) |
| 50-54 | 747401 (0.050) | 291574 (0.045) | 127836.5 (0.053) | | 5320 (711.8) | 2698 (925.3) | 1476 (1154.6) | 1237219.5 (0.080) | | 566942.5 (0.078) | 182901 (0.085) | | 6043 (488.4) | 3131 (552.3) | 1310 (716.2) | | 1266401.5 (0.083) | 689364.5 (0.084) | 191212 (0.087) | | 4422 (349.2) | 2696 (391.1) | 978 (511.5) |
| 55-59 | 612547.5 (0.041) | 258863.5 (0.040) | 138115 (0.058) | | 6653 (1086.1) | 3550 (1371.4) | 2294 (1660.9) | 857365.5 (0.055) | | 383434.5 (0.053) | 141557 (0.066) | | 6097 (711.1) | 3163 (824.9) | 1386 (979.1) | | 1249335 (0.082) | 674020.5 (0.082) | 213539.5 (0.097) | | 6571 (526.0) | 3839 (569.6) | 1532 (717.4) |
| 60-64 | 450655.5 (0.030) | 233285.5 (0.036) | 147252 (0.061) | | 7741 (1717.7) | 4727 (2026.3) | 3180 (2159.6) | 645135 (0.042) | | 296335.5 (0.041) | 127936.5 (0.059) | | 6709 (1039.9) | 3515 (1186.2) | 1665 (1301.4) | | 1028151 (0.067) | 550052.5 (0.067) | 199859 (0.091) | | 7776 (756.3) | 4651 (845.6) | 1792 (896.6) |
| 65-69 | 270437.5 (0.018) | 163757.5 (0.025) | 110232.5 (0.046) | | 7216 (2668.3) | 5014 (3061.8) | 3468 (3146.1) | 504285 (0.032) | | 247697.5 (0.034) | 125438 (0.058) | | 8589 (1703.2) | 4838 (1953.2) | 2496 (1989.8) | | 676898.5 (0.044) | 355048.5 (0.043) | 145993 (0.066) | | 7957 (1175.5) | 4434 (1248.8) | 2026 (1387.7) |
| 70-74 | 154839 (0.010) | 101504.5 (0.016) | 72612.5 (0.030) | | 6985 (4511.1) | 4953 (4879.6) | 3555 (4895.9) | 343695.5 (0.022) | | 198139.5 (0.027) | 115606.5 (0.054) | | 10211 (2970.9) | 6382 (3221.0) | 4045 (3498.9) | | 499294 (0.033) | 265726.5 (0.033) | 118730.5 (0.054) | | 9615 (1925.7) | 5492 (2066.8) | 2526 (2127.5) |
| 75-79 | 93715 (0.006) | 65368 (0.010) | 47693.5 (0.020) | | 6992 (7460.9) | 5166 (7902.9) | 3934 (8248.5) | 182272 (0.012) | | 116317 (0.016) | 71876.5 (0.033) | | 9269 (5085.3) | 6394 (5497.0) | 4103 (5708.4) | | 369908.5 (0.024) | 203440.5 (0.025) | 102496 (0.046) | | 13463 (3639.5) | 7878 (3872.4) | 4102 (4002.1) |
| 80-84 | 42844.5 (0.003) | 30906.5 (0.005) | 22276 (0.009) | | 5066 (11824.2) | 3842 (12431.0) | 2845 (12771.6) | 82417 (0.005) | | 53482 (0.007) | 34585.5 (0.016) | | 7182 (8714.2) | 4966 (9285.4) | 3295 (9527.1) | | 198681 (0.013) | 121110.5 (0.015) | 66979 (0.030) | | 13365 (6726.9) | 8729 (7207.5) | 4976 (7429.2) |
| 85-89 | 15291 (0.001) | 10554 (0.002) | 7750 (0.003) | | 2715 (17755.5) | 2028 (19215.5) | 1543 (19909.7) | 33651.5 (0.002) | | 22249.5 (0.003) | 14106.5 (0.007) | | 4994 (14840.3) | 3278 (14732.9) | 2217 (15716.2) | | 72139 (0.005) | 45997 (0.006) | 26272.5 (0.012) | | 8418 (11669.1) | 5686 (12361.7) | 3336 (12697.7) |
| 90-94 | 4009 (0.000) | 2665 (0.000) | 1857 (0.001) | | 1011 (25218.3) | 701 (26303.9) | 500 (26925.1) | 8480 (0.001) | | 5680.5 (0.001) | 3481 (0.002) | | 1915 (22582.5) | 1281 (22550.8) | 808 (23211.7) | | 18881.5 (0.001) | 11841.5 (0.001) | 6913 (0.003) | | 3694 (19564.1) | 2430 (20521.0) | 1400 (20251.7) |
| 95-99 | 424 (0.000) | 285 (0.000) | 221 (0.000) | | 136 (32075.5) | 131 (45964.9) | 102 (46153.8) | 1430 (0.000) | | 895 (0.000) | 543.5 (0.000) | | 420 (29370.6) | 270 (30167.6) | 168 (30910.8) | | 3948 (0.000) | 2462.5 (0.000) | 1261.5 (0.001) | | 1111 (28140.8) | 712 (28913.7) | 389 (30836.3) |
| 100+ | 65 (0.000) | 66 (0.000) | 48.5 (0.000) | | 27 (41538.5) | 30 (45454.5) | 24 (49484.5) | 185.5 (0.000) | | 130.5 (0.000) | 69 (0.000) | | 73 (39353.1) | 57 (43678.2) | 31 (44927.5) | | 353 (0.000) | 256 (0.000) | 126.5 (0.000) | | 157 (44475.9) | 79 (30859.4) | 42 (33201.6) |
| Total | 15017897.5 (1.000) | 6450776 (1.000) | 2394602 (1.000) | | 67158 (447.2) | 42921 (665.4) | 27706 (1157.0) | 15534643.5 (1.000) | | 7286022.5 (1.000) | 2156497.5 (1.000) | | 73991 (476.3) | 44284 (607.8) | 24039 (1114.7) | | 15233827 (1.000) | 8170072 (1.000) | 2205443 (1.000) | | 84389 (554.0) | 51378 (628.9) | 24509 (1111.3) |

In the Population column, proportions are shown in parentheses, whereas age-specific mortality (per 100,000 people) is shown in the death column. LUA=Large Urban Area; SMUA=Small-to-Middle Urban Area; RA=Rural Area.

Supplementary Table S4. Population and deaths by age group according to urban and rural classification in 2000, 2010, and 2019: Data from the Korean Statistical Information Services, Women.

|  | **2000** | | | | | | **2010** | | | | | | | **2019** | | | | | | |
| --- | --- | --- | --- | --- | --- | --- | --- | --- | --- | --- | --- | --- | --- | --- | --- | --- | --- | --- | --- | --- |
|  | **Population** | | | **Deaths** | | | **Population** | | | **Deaths** | | | | **Population** | | | | **Deaths** | | |
|  | **LMA** | **SMMA** | **RA** | **LMA** | **SMMA** | **RA** | **LMA** | **SMMA** | **RA** | **LMA** | **SMMA** | **RA** | **LMA** | | **SMMA** | **RA** | **LMA** | | **SMMA** | **RA** |
| 0 | 179824 (0.012) | 82184 (0.013) | 23042 (0.010) | 872 (484.9) | 348 (423.4) | 130 (564.2) | 128872.5 (0.008) | 67242 (0.009) | 15257.5 (0.007) | 370 (287.1) | 199 (295.9) | 51 (334.3) | 86638 (0.006) | | 51318.5 (0.006) | 11223.5 (0.005) | 201 (232.0) | | 124 (241.6) | 32 (285.1) |
| 1-4 | 792270 (0.053) | 371488 (0.058) | 103292 (0.043) | 297 (37.5) | 163 (43.9) | 78 (75.5) | 542386.5 (0.035) | 287778 (0.040) | 63233.5 (0.030) | 113 (20.8) | 52 (18.1) | 8 (12.7) | 450981.5 (0.029) | | 275079.5 (0.034) | 54730.5 (0.025) | 58 (12.9) | | 41 (14.9) | 12 (21.9) |
| 5-9 | 1050256 (0.070) | 478348.5 (0.075) | 130609.5 (0.055) | 183 (17.4) | 123 (25.7) | 51 (39.0) | 750662 (0.048) | 389869 (0.054) | 85496.5 (0.040) | 53 (7.1) | 40 (10.3) | 9 (10.5) | 651678.5 (0.042) | | 400030 (0.050) | 78456 (0.036) | 36 (5.5) | | 27 (6.7) | 7 (8.9) |
| 10-14 | 958886 (0.064) | 411744.5 (0.065) | 127809 (0.053) | 134 (14.0) | 72 (17.5) | 27 (21.1) | 979884 (0.063) | 478928 (0.067) | 108419 (0.051) | 93 (9.5) | 52 (10.9) | 18 (16.6) | 655886 (0.042) | | 383191 (0.048) | 78561 (0.036) | 38 (5.8) | | 32 (8.4) | 6 (7.6) |
| 15-19 | 1179639 (0.079) | 487642.5 (0.076) | 185988 (0.078) | 327 (27.7) | 172 (35.3) | 87 (46.8) | 1062933.5 (0.068) | 473034 (0.066) | 112502 (0.053) | 200 (18.8) | 95 (20.1) | 34 (30.2) | 789571 (0.051) | | 424002 (0.053) | 92783.5 (0.043) | 139 (17.6) | | 79 (18.6) | 18 (19.4) |
| 20-24 | 1256036 (0.084) | 482623 (0.076) | 175546 (0.073) | 424 (33.8) | 221 (45.8) | 118 (67.2) | 984576 (0.063) | 404353 (0.056) | 100071 (0.047) | 325 (33.0) | 130 (32.2) | 42 (42.0) | 1028754.5 (0.066) | | 469306.5 (0.058) | 96556 (0.045) | 273 (26.5) | | 141 (30.0) | 30 (31.1) |
| 25-29 | 1484290 (0.100) | 557862 (0.087) | 151215 (0.063) | 592 (39.9) | 302 (54.1) | 113 (74.7) | 1257677 (0.081) | 481440.5 (0.067) | 108657 (0.051) | 601 (47.8) | 215 (44.7) | 63 (58.0) | 1090258 (0.070) | | 446107 (0.056) | 83197.5 (0.038) | 339 (31.1) | | 140 (31.4) | 18 (21.6) |
| 30-34 | 1443792 (0.097) | 597698 (0.094) | 151452 (0.063) | 761 (52.7) | 435 (72.8) | 188 (124.1) | 1258852.5 (0.081) | 538830.5 (0.075) | 110889 (0.052) | 649 (51.6) | 298 (55.3) | 76 (68.5) | 984329.5 (0.063) | | 454042.5 (0.057) | 85201 (0.039) | 403 (40.9) | | 216 (47.6) | 37 (43.4) |
| 35-39 | 1416125.5 (0.095) | 574437.5 (0.090) | 155365.5 (0.065) | 1051 (74.2) | 573 (99.7) | 234 (150.6) | 1407761.5 (0.090) | 636108 (0.089) | 127564 (0.060) | 946 (67.2) | 478 (75.1) | 129 (101.1) | 1201581 (0.077) | | 623675 (0.078) | 116703.5 (0.054) | 666 (55.4) | | 381 (61.1) | 80 (68.5) |
| 40-44 | 1338055.5 (0.090) | 498487.5 (0.078) | 153397.5 (0.064) | 1558 (116.4) | 723 (145.0) | 297 (193.6) | 1409858 (0.090) | 619556.5 (0.086) | 136339 (0.064) | 1204 (85.4) | 604 (97.5) | 180 (132.0) | 1165788 (0.075) | | 613292 (0.076) | 118340 (0.055) | 817 (70.1) | | 521 (85.0) | 126 (106.5) |
| 45-49 | 947597 (0.064) | 346741 (0.054) | 132073.5 (0.055) | 1538 (162.3) | 711 (205.1) | 314 (237.7) | 1370326 (0.088) | 589502.5 (0.082) | 155965.5 (0.073) | 1756 (128.1) | 836 (141.8) | 302 (193.6) | 1373947 (0.088) | | 687888 (0.086) | 142863 (0.066) | 1368 (99.6) | | 820 (119.2) | 185 (129.5) |
| 50-54 | 736899.5 (0.049) | 283525.5 (0.044) | 131465.5 (0.055) | 1812 (245.9) | 901 (317.8) | 450 (342.3) | 1255780.5 (0.081) | 534930 (0.075) | 167618.5 (0.078) | 2223 (177.0) | 978 (182.8) | 432 (257.7) | 1302717 (0.084) | | 649671.5 (0.081) | 159488.5 (0.074) | 1731 (132.9) | | 980 (150.8) | 289 (181.2) |
| 55-59 | 617024 (0.041) | 292608 (0.046) | 169354.5 (0.071) | 2498 (404.8) | 1377 (470.6) | 827 (488.3) | 876802.5 (0.056) | 379363.5 (0.053) | 142800.5 (0.067) | 2062 (235.2) | 1055 (278.1) | 424 (296.9) | 1283296.5 (0.083) | | 645130.5 (0.080) | 194569.5 (0.090) | 2493 (194.3) | | 1299 (201.4) | 461 (236.9) |
| 60-64 | 496739 (0.033) | 284255.5 (0.045) | 184517 (0.077) | 3409 (686.3) | 2059 (724.3) | 1342 (727.3) | 677314 (0.043) | 307515.5 (0.043) | 134230 (0.063) | 2578 (380.6) | 1358 (441.6) | 572 (426.1) | 1087057.5 (0.070) | | 546501.5 (0.068) | 189831.5 (0.088) | 2798 (257.4) | | 1649 (301.7) | 595 (313.4) |
| 65-69 | 369298.5 (0.025) | 233215 (0.037) | 154152.5 (0.064) | 4616 (1249.9) | 3055 (1310.0) | 1880 (1219.6) | 564131 (0.036) | 301672 (0.042) | 161034.5 (0.075) | 3882 (688.1) | 2351 (779.3) | 1215 (754.5) | 740400.5 (0.048) | | 374909.5 (0.047) | 150589.5 (0.069) | 3244 (438.1) | | 1756 (468.4) | 764 (507.3) |
| 70-74 | 272757 (0.018) | 169447.5 (0.027) | 112314 (0.047) | 6790 (2489.4) | 4223 (2492.2) | 2643 (2353.2) | 444729.5 (0.029) | 272042 (0.038) | 162840.5 (0.076) | 5980 (1344.6) | 3766 (1384.3) | 2157 (1324.6) | 581788 (0.037) | | 302469 (0.038) | 134224 (0.062) | 4667 (802.2) | | 2582 (853.6) | 1201 (894.8) |
| 75-79 | 187146.5 (0.013) | 116094 (0.018) | 77047.5 (0.032) | 8576 (4582.5) | 5292 (4558.4) | 3546 (4602.4) | 306974 (0.020) | 199674.5 (0.028) | 122474.5 (0.057) | 8640 (2814.6) | 5405 (2706.9) | 3176 (2593.2) | 483940.5 (0.031) | | 288627.5 (0.036) | 154832.5 (0.071) | 8488 (1753.9) | | 5376 (1862.6) | 2901 (1873.6) |
| 80-84 | 104190.5 (0.007) | 65664 (0.010) | 43618 (0.018) | 8911 (8552.6) | 5683 (8654.7) | 3701 (8485.0) | 189997.5 (0.012) | 119838 (0.017) | 73121 (0.034) | 10584 (5570.6) | 6543 (5459.9) | 3821 (5225.6) | 327452 (0.021) | | 215899.5 (0.027) | 126043 (0.058) | 12738 (3890.0) | | 8461 (3919.0) | 4871 (3864.6) |
| 85-89 | 49160.5 (0.003) | 31826.5 (0.005) | 21510.5 (0.009) | 6821 (13875.0) | 4434 (13931.8) | 3143 (14611.5) | 92166.5 (0.006) | 58842.5 (0.008) | 35641 (0.017) | 9449 (10252.1) | 5993 (10184.8) | 3452 (9685.5) | 169732 (0.011) | | 117206.5 (0.015) | 68272 (0.032) | 14121 (8319.6) | | 9479 (8087.4) | 5326 (7801.1) |
| 90-94 | 15096.5 (0.001) | 10353.5 (0.002) | 7101 (0.003) | 3223 (21349.3) | 2262 (21847.7) | 1614 (22729.2) | 29506 (0.002) | 19300 (0.003) | 11595 (0.005) | 5211 (17660.8) | 3215 (16658.0) | 2123 (18309.6) | 64644.5 (0.004) | | 42815 (0.005) | 24123 (0.011) | 9966 (15416.6) | | 6394 (14934.0) | 3579 (14836.5) |
| 95-99 | 2983 (0.000) | 2111 (0.000) | 1521.5 (0.001) | 850 (28494.8) | 658 (31170.1) | 507 (33322.4) | 6563.5 (0.000) | 4435 (0.001) | 2636 (0.001) | 1675 (25519.9) | 1123 (25321.3) | 711 (26972.7) | 14941.5 (0.001) | | 10606.5 (0.001) | 5849.5 (0.003) | 3715 (24863.6) | | 2592 (24437.8) | 1409 (24087.5) |
| 100+ | 874.5 (0.000) | 662.5 (0.000) | 489.5 (0.000) | 238 (27215.6) | 186 (28075.5) | 191 (39019.4) | 1072 (0.000) | 733.5 (0.000) | 447.5 (0.000) | 272 (25373.1) | 242 (32992.5) | 132 (29497.2) | 2125.5 (0.000) | | 1501 (0.000) | 864.5 (0.000) | 687 (32321.8) | | 463 (30846.1) | 277 (32041.6) |
| Total | 14898940.5 (1.000) | 6379019.5 (1.000) | 2392881.5 (1.000) | 55481 (372.4) | 33973 (532.6) | 21481 (897.7) | 15598826.5 (1.000) | 7164988.5 (1.000) | 2138833 (1.000) | 58866 (377.4) | 35028 (488.9) | 19127 (894.3) | 15537508.5 (1.000) | | 8023270 (1.000) | 2167303 (1.000) | 68986 (444.0) | | 43553 (542.8) | 22224 (1025.4) |

In the Population column, proportions are shown in parentheses, whereas age-specific mortality (per 100,000 people) is shown in the death column. LUA=Large Urban Area; SMUA=Small-to-Middle Urban Area; RA=Rural Area.

**
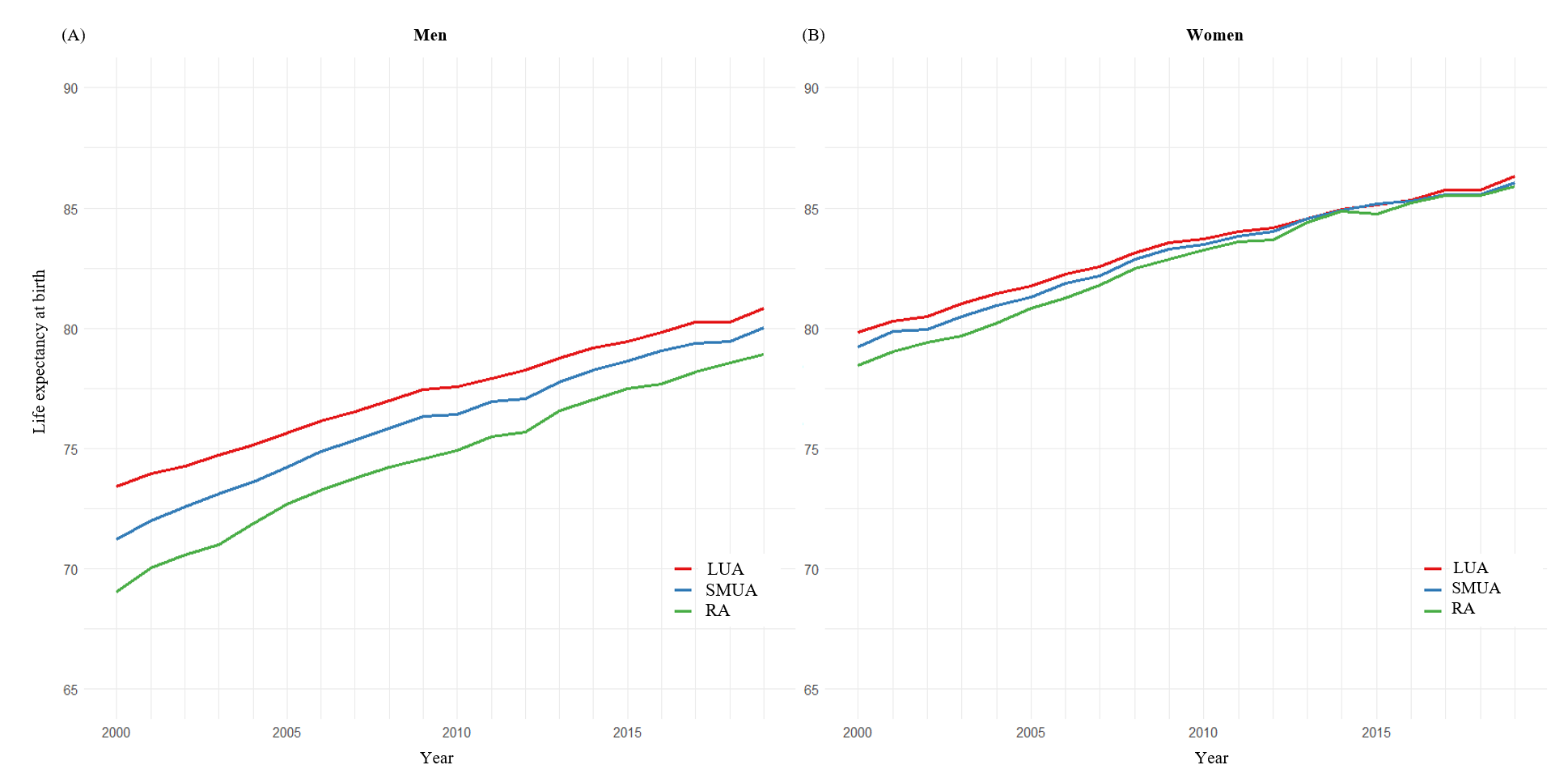
**

Supplementary Figure S1. Annual trends of life expectancy by degree of urbanization in Korea, 2000−2019: Findings from the Korean Statistical Information Service, Men and women

(A) Annual trends of life expectancy by degree of urbanization, men (B) Annual trends of life expectancy by degree of urbanization, women

LUA = Large Urban Areas; SMUA = Small-to-Middle Urban Areas; RA = Rural Areas.

Supplementary Table S5. Point estimate and 95% uncertainty interval of the contour decomposition analysis at the starting point of each decennial period: Findings from the Korean Statistical Information Services, Men and women.

| **Causes of death / Age group** | **Year 2000** | | | | | **Year 2010** | | | | |
| --- | --- | --- | --- | --- | --- | --- | --- | --- | --- | --- |
|  | **0-14** | **15-39** | **40-64** | **65-79** | **80+** | **0-14** | **15-39** | **40-64** | **65-79** | **80+** |
| **Men** |  |  |  |  |  |  |  |  |  |  |
| Certain infectious and parasitic diseases | 0.03 (0.01,0.05) | 0.03 (0.02,0.05) | 0.08 (0.05,0.11) | 0.01 (-0.01,0.03) | -0.01 (-0.03,0.01) | 0.00 (-0.01,0.02) | 0.01 (0.00,0.03) | 0.05 (0.03,0.08) | 0.01 (0.00,0.03) | 0.01 (-0.01,0.04) |
| Neoplasms | 0.00 (-0.02,0.02) | 0.10 (0.07,0.14) | 0.46 (0.38,0.54) | 0.13 (0.06,0.20) | -0.03 (-0.08,0.01) | 0.01 (-0.01,0.04) | 0.03 (0.00,0.06) | 0.28 (0.21,0.35) | 0.26 (0.20,0.33) | 0.02 (-0.04,0.07) |
| Diseases of the blood and blood-forming organs and certain disorders involving the immune mechanism | 0.00 (-0.01,0.01) | 0.00 (0.00,0.01) | 0.00 (0.00,0.00) | -0.01 (-0.01,0.00) | 0.00 (0.00,0.01) | 0.00 (-0.01,0.00) | 0.00 (0.00,0.01) | 0.00 (0.00,0.01) | 0.00 (0.00,0.01) | 0.00 (-0.01,0.01) |
| Endocrine, nutritional, and metabolic diseases | -0.01 (-0.01,-0.01) | 0.02 (0.01,0.03) | 0.09 (0.06,0.13) | -0.10 (-0.13,-0.08) | -0.06 (-0.07,-0.04) | 0.01 (0.00,0.03) | 0.00 (0.00,0.01) | 0.08 (0.06,0.11) | -0.01 (-0.04,0.01) | -0.02 (-0.04,0.00) |
| Mental and behavioral disorders | 0.00 (0.00,0.00) | 0.04 (0.03,0.06) | 0.08 (0.05,0.10) | 0.01 (-0.01,0.02) | -0.01 (-0.03,0.01) | 0.00 (0.00,0.00) | 0.01 (0.00,0.02) | 0.02 (0.00,0.04) | 0.00 (-0.01,0.01) | -0.02 (-0.04,0.00) |
| Diseases of the nervous system | 0.00 (-0.02,0.02) | 0.03 (0.01,0.04) | 0.01 (0.00,0.03) | -0.01 (-0.02,0.00) | -0.01 (-0.02,0.00) | 0.01 (-0.01,0.04) | 0.02 (0.00,0.03) | 0.01 (0.00,0.03) | -0.03 (-0.04,-0.01) | -0.02 (-0.04,0.00) |
| Diseases of the circulatory system | 0.01 (-0.01,0.03) | 0.09 (0.06,0.12) | 0.24 (0.18,0.31) | -0.03 (-0.09,0.03) | 0.02 (-0.03,0.07) | 0.00 (-0.01,0.02) | 0.03 (0.01,0.06) | 0.16 (0.11,0.21) | 0.01 (-0.04,0.06) | 0.01 (-0.04,0.06) |
| Diseases of the respiratory system | 0.01 (-0.01,0.03) | 0.03 (0.01,0.04) | 0.14 (0.11,0.18) | 0.09 (0.05,0.13) | 0.01 (-0.03,0.05) | -0.01 (-0.02,0.01) | 0.01 (0.00,0.01) | 0.07 (0.04,0.09) | 0.09 (0.06,0.12) | 0.07 (0.02,0.12) |
| Diseases of the digestive system | 0.00 (0.00,0.01) | 0.10 (0.08,0.13) | 0.56 (0.50,0.63) | 0.14 (0.10,0.16) | 0.02 (0.00,0.05) | 0.01 (0.00,0.02) | 0.03 (0.01,0.05) | 0.24 (0.20,0.28) | 0.05 (0.03,0.07) | 0.02 (0.00,0.04) |
| Diseases of the musculoskeletal system and connective tissue | 0.00 (0.00,0.00) | 0.01 (0.00,0.01) | 0.01 (0.00,0.01) | 0.03 (0.02,0.04) | 0.01 (0.00,0.02) | 0.00 (0.00,0.00) | 0.00 (0.00,0.01) | 0.01 (0.00,0.02) | 0.00 (-0.01,0.01) | 0.00 (-0.01,0.01) |
| Diseases of the genitourinary system | 0.00 (0.00,0.00) | 0.01 (0.00,0.02) | -0.01 (-0.02,0.01) | -0.03 (-0.04,-0.01) | -0.01 (-0.02,0.00) | 0.00 (0.00,0.00) | 0.00 (0.00,0.01) | 0.01 (0.00,0.03) | -0.01 (-0.02,0.01) | -0.03 (-0.05,-0.01) |
| Symptoms, signs, and abnormal clinical and laboratory findings | 0.05 (0.02,0.09) | 0.02 (0.00,0.04) | 0.10 (0.07,0.13) | 0.27 (0.23,0.31) | 0.26 (0.21,0.30) | 0.00 (-0.02,0.04) | 0.04 (0.01,0.06) | 0.03 (-0.01,0.06) | 0.05 (0.03,0.08) | 0.09 (0.05,0.13) |
| External causes of mortality | 0.25 (0.18,0.31) | 0.91 (0.82,1.00) | 0.81 (0.74,0.89) | 0.12 (0.09,0.15) | 0.00 (-0.02,0.01) | 0.10 (0.05,0.15) | 0.33 (0.25,0.41) | 0.54 (0.48,0.61) | 0.25 (0.22,0.29) | 0.09 (0.07,0.12) |
| Others | 0.06 (0.00,0.13) | 0.00 (0.00,0.01) | 0.00 (0.00,0.01) | 0.00 (0.00,0.01) | 0.00 (-0.01,0.00) | 0.02 (-0.04,0.10) | 0.01 (0.00,0.02) | 0.00 (0.00,0.01) | 0.00 (-0.01,0.00) | 0.01 (0.00,0.01) |
| **Women** |  |  |  |  |  |  |  |  |  |  |
| Certain infectious and parasitic diseases | 0.01 (-0.01,0.03) | 0.01 (0.00,0.03) | 0.02 (0.00,0.04) | 0.02 (0.00,0.04) | -0.02 (-0.04,-0.01) | -0.01 (-0.01,0.00) | 0.00 (-0.01,0.01) | 0.02 (0.00,0.04) | 0.00 (-0.02,0.01) | 0.01 (-0.01,0.03) |
| Neoplasms | 0.02 (0.00,0.05) | 0.07 (0.03,0.11) | -0.02 (-0.08,0.05) | -0.10 (-0.15,-0.05) | -0.11 (-0.15,-0.08) | 0.03 (0.00,0.06) | 0.07 (0.03,0.11) | 0.07 (0.01,0.14) | -0.08 (-0.13,-0.03) | -0.04 (-0.08,0.00) |
| Diseases of the blood and blood-forming organs and certain disorders involving the immune mechanism | -0.01 (-0.01,0.00) | 0.00 (-0.01,0.00) | 0.00 (-0.01,0.01) | 0.00 (0.00,0.01) | 0.00 (-0.01,0.00) | 0.00 (-0.01,0.00) | 0.00 (-0.01,0.01) | 0.00 (-0.01,0.00) | 0.00 (0.00,0.01) | -0.01 (-0.01,0.00) |
| Endocrine, nutritional, and metabolic diseases | 0.00 (-0.01,0.00) | 0.02 (0.00,0.03) | 0.01 (-0.01,0.03) | -0.12 (-0.15,-0.09) | -0.07 (-0.09,-0.05) | 0.00 (-0.01,0.00) | 0.00 (-0.01,0.01) | 0.01 (-0.01,0.03) | -0.06 (-0.08,-0.03) | -0.05 (-0.07,-0.02) |
| Mental and behavioral disorders | 0.00 (0.00,0.00) | 0.02 (0.00,0.03) | 0.02 (0.01,0.03) | 0.00 (-0.02,0.02) | 0.00 (-0.04,0.03) | 0.00 (0.00,0.00) | 0.00 (0.00,0.01) | 0.01 (0.00,0.02) | 0.01 (-0.01,0.02) | 0.00 (-0.03,0.02) |
| Diseases of the nervous system | 0.02 (-0.01,0.04) | 0.04 (0.02,0.06) | 0.00 (-0.01,0.02) | -0.02 (-0.03,0.00) | -0.01 (-0.03,0.00) | 0.01 (-0.01,0.03) | 0.02 (0.00,0.04) | 0.02 (0.00,0.03) | -0.02 (-0.04,-0.01) | -0.03 (-0.05,0.00) |
| Diseases of the circulatory system | -0.01 (-0.02,0.01) | 0.08 (0.05,0.11) | 0.19 (0.14,0.25) | -0.13 (-0.19,-0.06) | -0.01 (-0.08,0.05) | -0.01 (-0.01,0.00) | 0.00 (-0.01,0.02) | 0.11 (0.07,0.14) | 0.03 (-0.02,0.07) | -0.11 (-0.18,-0.05) |
| Diseases of the respiratory system | 0.03 (0.01,0.06) | 0.02 (0.00,0.03) | 0.01 (-0.01,0.03) | 0.02 (-0.01,0.06) | -0.04 (-0.08,0.00) | 0.00 (-0.02,0.01) | 0.01 (0.00,0.02) | 0.02 (0.00,0.03) | 0.00 (-0.02,0.03) | 0.04 (0.00,0.09) |
| Diseases of the digestive system | 0.01 (0.00,0.02) | 0.03 (0.01,0.05) | 0.05 (0.03,0.08) | 0.00 (-0.02,0.02) | 0.00 (-0.02,0.03) | 0.00 (-0.01,0.02) | 0.01 (0.00,0.03) | 0.04 (0.02,0.07) | -0.01 (-0.02,0.01) | 0.00 (-0.02,0.02) |
| Diseases of the musculoskeletal system and connective tissue | 0.00 (0.00,0.00) | 0.00 (0.00,0.01) | 0.01 (0.00,0.02) | 0.02 (0.01,0.04) | 0.00 (-0.02,0.02) | 0.00 (0.00,0.00) | 0.00 (-0.01,0.01) | 0.00 (-0.01,0.01) | 0.00 (-0.01,0.01) | 0.01 (-0.01,0.02) |
| Diseases of the genitourinary system | 0.00 (0.00,0.00) | 0.00 (-0.01,0.00) | -0.01 (-0.02,0.01) | -0.04 (-0.05,-0.02) | -0.04 (-0.05,-0.02) | 0.00 (0.00,0.00) | 0.00 (0.00,0.01) | 0.03 (0.01,0.04) | -0.03 (-0.04,-0.02) | -0.03 (-0.05,-0.01) |
| Pregnancy, childbirth, and the puerperium | 0.00 (0.00,0.00) | 0.00 (0.00,0.01) | 0.00 (0.00,0.00) | 0.00 (0.00,0.00) | 0.00 (0.00,0.00) | 0.00 (0.00,0.00) | 0.00 (-0.01,0.01) | 0.00 (0.00,0.00) | 0.00 (0.00,0.00) | 0.00 (0.00,0.00) |
| Symptoms, signs, and abnormal clinical and laboratory findings | 0.05 (0.01,0.10) | 0.02 (0.00,0.04) | 0.04 (0.02,0.06) | 0.18 (0.14,0.22) | 0.36 (0.30,0.42) | 0.01 (-0.02,0.05) | 0.01 (-0.01,0.03) | 0.02 (0.00,0.03) | -0.01 (-0.03,0.01) | 0.01 (-0.04,0.06) |
| External causes of mortality | 0.14 (0.09,0.20) | 0.32 (0.26,0.38) | 0.27 (0.23,0.32) | 0.06 (0.03,0.08) | -0.01 (-0.03,0.01) | 0.02 (-0.02,0.06) | 0.10 (0.03,0.17) | 0.18 (0.13,0.23) | 0.09 (0.07,0.12) | 0.05 (0.02,0.08) |
| Others | 0.02 (-0.04,0.10) | 0.01 (0.00,0.02) | 0.00 (-0.01,0.00) | 0.00 (0.00,0.01) | 0.00 (-0.01,0.01) | 0.01 (-0.06,0.09) | 0.00 (0.00,0.02) | 0.00 (0.00,0.01) | 0.01 (0.00,0.01) | 0.01 (0.00,0.02) |

The numbers in parentheses indicate the boundary values of the 95% uncertainty interval.

Supplementary Table S6. Point estimate and 95% uncertainty interval of the contour decomposition analysis of changes in life expectancy at birth for each period: Findings from the Korean Statistical Information Services, Men and women.

| **Causes of death / Age group** | **Period 2000-2009** | | | | | | **Period 2010-2019** | | | | |
| --- | --- | --- | --- | --- | --- | --- | --- | --- | --- | --- | --- |
|  | **0-14** | **15-39** | **40-64** | **65-79** | **80+** | **0-14** | | **15-39** | **40-64** | **65-79** | **80+** |
| **Men** |  |  |  |  |  |  | |  |  |  |  |
| Certain infectious and parasitic diseases | -0.04 (-0.06,-0.02) | -0.03 (-0.05,-0.01) | -0.05 (-0.08,-0.02) | 0.02 (-0.01,0.04) | 0.01 (-0.01,0.03) | -0.01 (-0.03,0.01) | | -0.02 (-0.04,-0.01) | -0.03 (-0.06,0.00) | 0.01 (-0.01,0.03) | 0.01 (-0.02,0.03) |
| Neoplasms | 0.01 (-0.02,0.04) | -0.09 (-0.14,-0.05) | -0.22 (-0.32,-0.12) | 0.10 (0.01,0.19) | 0.01 (-0.04,0.06) | -0.02 (-0.05,0.01) | | -0.01 (-0.06,0.03) | -0.10 (-0.19,-0.01) | -0.23 (-0.31,-0.14) | 0.02 (-0.04,0.09) |
| Diseases of the blood and blood-forming organs and certain disorders involving the immune mechanism | 0.00 (-0.01,0.01) | 0.00 (-0.01,0.01) | 0.00 (-0.01,0.01) | 0.00 (0.00,0.01) | 0.00 (-0.01,0.00) | 0.00 (0.00,0.01) | | 0.00 (-0.01,0.01) | 0.00 (-0.01,0.01) | 0.00 (-0.01,0.00) | 0.00 (-0.01,0.01) |
| Endocrine, nutritional, and metabolic diseases | 0.01 (0.00,0.02) | 0.00 (-0.02,0.01) | -0.01 (-0.05,0.03) | 0.07 (0.04,0.10) | 0.03 (0.01,0.06) | -0.01 (-0.03,0.01) | | -0.01 (-0.02,0.01) | -0.07 (-0.10,-0.03) | 0.01 (-0.02,0.03) | 0.02 (0.00,0.05) |
| Mental and behavioral disorders | 0.00 (0.00,0.00) | -0.03 (-0.05,-0.01) | -0.05 (-0.08,-0.02) | 0.00 (-0.02,0.02) | 0.01 (-0.01,0.04) | 0.00 (0.00,0.00) | | 0.00 (-0.02,0.01) | 0.00 (-0.02,0.02) | 0.00 (-0.02,0.01) | 0.03 (0.01,0.05) |
| Diseases of the nervous system | 0.00 (-0.02,0.03) | 0.01 (-0.01,0.04) | 0.01 (-0.01,0.03) | 0.00 (-0.01,0.02) | 0.01 (-0.01,0.02) | 0.00 (-0.03,0.03) | | 0.00 (-0.03,0.02) | 0.00 (-0.01,0.02) | 0.01 (-0.01,0.02) | -0.02 (-0.04,0.01) |
| Diseases of the circulatory system | -0.01 (-0.04,0.01) | -0.07 (-0.11,-0.03) | -0.06 (-0.14,0.02) | 0.00 (-0.07,0.07) | -0.02 (-0.08,0.05) | 0.00 (-0.03,0.02) | | -0.01 (-0.05,0.02) | -0.08 (-0.14,-0.02) | -0.01 (-0.07,0.05) | 0.05 (-0.01,0.11) |
| Diseases of the respiratory system | -0.01 (-0.03,0.02) | -0.01 (-0.03,0.01) | -0.09 (-0.12,-0.05) | -0.01 (-0.05,0.04) | 0.04 (0.00,0.09) | 0.02 (0.00,0.04) | | 0.01 (0.00,0.03) | 0.02 (-0.01,0.05) | 0.05 (0.01,0.10) | 0.06 (-0.01,0.12) |
| Diseases of the digestive system | -0.01 (-0.02,0.00) | -0.07 (-0.10,-0.03) | -0.34 (-0.41,-0.27) | -0.10 (-0.14,-0.07) | -0.01 (-0.03,0.02) | -0.01 (-0.02,0.00) | | -0.02 (-0.05,0.00) | -0.12 (-0.18,-0.07) | -0.02 (-0.05,0.01) | -0.01 (-0.03,0.02) |
| Diseases of the musculoskeletal system and connective tissue | 0.00 (0.00,0.00) | 0.00 (-0.01,0.00) | 0.00 (-0.01,0.01) | -0.02 (-0.03,-0.01) | 0.00 (-0.01,0.02) | 0.00 (0.00,0.00) | | 0.00 (-0.01,0.00) | 0.00 (-0.01,0.01) | 0.01 (0.00,0.02) | 0.00 (-0.01,0.01) |
| Diseases of the genitourinary system | 0.01 (0.00,0.02) | 0.00 (-0.02,0.01) | 0.02 (0.00,0.04) | 0.02 (0.00,0.04) | 0.00 (-0.02,0.02) | 0.00 (0.00,0.00) | | 0.00 (-0.01,0.01) | -0.01 (-0.03,0.01) | 0.02 (0.00,0.04) | 0.02 (0.00,0.04) |
| Symptoms, signs, and abnormal clinical and laboratory findings | -0.02 (-0.07,0.02) | 0.01 (-0.02,0.04) | -0.08 (-0.11,-0.04) | -0.21 (-0.25,-0.17) | -0.15 (-0.21,-0.10) | -0.02 (-0.06,0.03) | | -0.01 (-0.04,0.03) | 0.11 (0.06,0.15) | -0.02 (-0.05,0.02) | -0.11 (-0.16,-0.06) |
| External causes of mortality | -0.19 (-0.27,-0.11) | -0.45 (-0.57,-0.33) | -0.32 (-0.41,-0.22) | 0.07 (0.03,0.12) | 0.04 (0.01,0.07) | -0.06 (-0.13,0.01) | | -0.16 (-0.27,-0.05) | -0.29 (-0.38,-0.21) | -0.12 (-0.16,-0.07) | -0.04 (-0.07,-0.01) |
| Others | -0.05 (-0.14,0.05) | 0.00 (-0.01,0.00) | 0.00 (0.00,0.01) | 0.00 (-0.01,0.00) | 0.00 (-0.01,0.01) | -0.02 (-0.12,0.07) | | -0.01 (-0.02,0.00) | 0.00 (-0.01,0.00) | 0.01 (0.00,0.01) | -0.01 (-0.02,0.00) |
| **Women** |  |  |  |  |  |  | |  |  |  |  |
| Certain infectious and parasitic diseases | -0.01 (-0.03,0.01) | 0.00 (-0.02,0.02) | -0.01 (-0.03,0.02) | -0.01 (-0.04,0.01) | 0.02 (0.00,0.04) | 0.01 (0.00,0.03) | | -0.01 (-0.02,0.00) | 0.00 (-0.02,0.03) | 0.03 (0.00,0.05) | 0.03 (0.00,0.06) |
| Neoplasms | -0.03 (-0.07,0.01) | -0.01 (-0.06,0.05) | 0.06 (-0.03,0.14) | -0.01 (-0.08,0.06) | 0.07 (0.02,0.12) | -0.02 (-0.06,0.01) | | -0.03 (-0.09,0.02) | 0.00 (-0.08,0.09) | 0.04 (-0.02,0.11) | -0.04 (-0.10,0.01) |
| Diseases of the blood and blood-forming organs and certain disorders involving the immune mechanism | 0.01 (0.00,0.02) | 0.00 (0.00,0.01) | 0.00 (0.00,0.01) | 0.00 (-0.01,0.01) | 0.00 (0.00,0.01) | 0.00 (0.00,0.00) | | 0.00 (-0.01,0.00) | 0.01 (0.00,0.01) | 0.00 (-0.01,0.00) | 0.01 (0.00,0.02) |
| Endocrine, nutritional, and metabolic diseases | 0.00 (-0.01,0.01) | -0.01 (-0.03,0.01) | 0.01 (-0.02,0.04) | 0.09 (0.05,0.12) | 0.03 (0.00,0.06) | 0.01 (0.00,0.03) | | 0.00 (-0.01,0.01) | 0.00 (-0.02,0.02) | 0.07 (0.04,0.09) | 0.02 (-0.01,0.05) |
| Mental and behavioral disorders | 0.00 (0.00,0.01) | -0.01 (-0.02,0.01) | -0.01 (-0.03,0.01) | 0.00 (-0.02,0.03) | 0.01 (-0.03,0.04) | 0.00 (0.00,0.00) | | 0.00 (-0.01,0.01) | 0.00 (-0.02,0.01) | 0.00 (-0.01,0.01) | -0.03 (-0.06,0.00) |
| Diseases of the nervous system | -0.01 (-0.05,0.02) | -0.02 (-0.04,0.01) | 0.02 (0.00,0.04) | 0.01 (-0.01,0.02) | 0.01 (-0.01,0.04) | 0.00 (-0.03,0.03) | | -0.01 (-0.03,0.01) | -0.02 (-0.04,0.00) | 0.01 (-0.01,0.03) | -0.04 (-0.07,0.00) |
| Diseases of the circulatory system | 0.01 (-0.02,0.03) | -0.07 (-0.10,-0.03) | -0.10 (-0.16,-0.03) | 0.12 (0.04,0.20) | -0.01 (-0.10,0.07) | 0.00 (-0.01,0.02) | | 0.01 (-0.02,0.04) | -0.05 (-0.10,0.00) | 0.05 (-0.01,0.10) | 0.14 (0.06,0.22) |
| Diseases of the respiratory system | -0.03 (-0.06,0.01) | 0.00 (-0.02,0.02) | 0.00 (-0.02,0.02) | -0.01 (-0.05,0.03) | 0.07 (0.02,0.12) | 0.00 (-0.02,0.01) | | -0.01 (-0.02,0.01) | 0.01 (-0.01,0.03) | 0.04 (0.01,0.07) | 0.04 (-0.02,0.09) |
| Diseases of the digestive system | 0.00 (-0.02,0.02) | -0.01 (-0.03,0.01) | -0.03 (-0.06,0.01) | 0.00 (-0.02,0.03) | 0.00 (-0.02,0.03) | 0.00 (-0.02,0.00) | | -0.01 (-0.03,0.02) | 0.00 (-0.03,0.03) | 0.02 (-0.01,0.04) | 0.01 (-0.02,0.03) |
| Diseases of the musculoskeletal system and connective tissue | 0.00 (0.00,0.00) | 0.00 (-0.01,0.00) | -0.01 (-0.02,0.00) | -0.01 (-0.03,0.00) | 0.03 (0.01,0.05) | 0.00 (0.00,0.00) | | 0.00 (-0.01,0.02) | 0.01 (0.00,0.02) | 0.01 (0.00,0.02) | -0.01 (-0.03,0.00) |
| Diseases of the genitourinary system | 0.01 (0.00,0.02) | 0.01 (0.00,0.03) | 0.02 (0.00,0.04) | 0.01 (-0.01,0.03) | 0.01 (-0.01,0.03) | 0.00 (0.00,0.00) | | 0.00 (-0.01,0.01) | -0.02 (-0.04,0.00) | 0.04 (0.02,0.06) | 0.03 (0.00,0.05) |
| Pregnancy, childbirth, and the puerperium | 0.00 (0.00,0.00) | -0.01 (-0.02,0.00) | 0.00 (0.00,0.00) | 0.00 (0.00,0.00) | 0.00 (0.00,0.00) | 0.00 (0.00,0.00) | | 0.00 (-0.01,0.02) | 0.00 (0.00,0.00) | 0.00 (0.00,0.00) | 0.00 (0.00,0.00) |
| Symptoms, signs, and abnormal clinical and laboratory findings | -0.03 (-0.08,0.02) | 0.00 (-0.02,0.03) | -0.03 (-0.05,0.00) | -0.14 (-0.18,-0.09) | -0.27 (-0.34,-0.19) | -0.01 (-0.06,0.05) | | -0.01 (-0.04,0.01) | 0.02 (-0.01,0.04) | 0.04 (0.01,0.07) | -0.14 (-0.20,-0.07) |
| External causes of mortality | -0.13 (-0.20,-0.06) | -0.21 (-0.30,-0.11) | -0.12 (-0.18,-0.06) | 0.01 (-0.02,0.05) | 0.04 (0.01,0.07) | 0.00 (-0.05,0.06) | | -0.14 (-0.23,-0.05) | -0.12 (-0.18,-0.06) | -0.05 (-0.08,-0.02) | -0.04 (-0.07,-0.01) |
| Others | 0.04 (-0.07,0.14) | -0.01 (-0.02,0.00) | 0.00 (0.00,0.01) | 0.00 (-0.01,0.00) | 0.00 (-0.01,0.01) | 0.05 (-0.06,0.16) | | 0.00 (-0.02,0.01) | 0.00 (0.00,0.01) | 0.00 (-0.01,0.00) | 0.00 (-0.01,0.01) |

The numbers in parentheses indicate the boundary values of the 95% uncertainty interval.

Supplementary Table S7. Point estimate and 95% uncertainty interval of contour decomposition analysis at the end point of each decennial period: Findings from the Korean Statistical Information Services, Men, and women.

| **Causes of death / Age group** | **Year 2009** | | | | | | **Year 2019** | | | | |
| --- | --- | --- | --- | --- | --- | --- | --- | --- | --- | --- | --- |
|  | **0-14** | **15-39** | **40-64** | **65-79** | **80+** | **0-14** | | **15-39** | **40-64** | **65-79** | **80+** |
| **Men** |  |  |  |  |  |  | |  |  |  |  |
| Certain infectious and parasitic diseases | -0.01 (-0.01,0.00) | 0.00 (-0.01,0.01) | 0.03 (0.01,0.05) | 0.03 (0.01,0.04) | 0.00 (-0.01,0.01) | 0.00 (-0.01,0.01) | | -0.01 (-0.01,0.00) | 0.02 (0.01,0.04) | 0.03 (0.01,0.04) | 0.02 (0.01,0.03) |
| Neoplasms | 0.01 (-0.02,0.03) | 0.01 (-0.02,0.04) | 0.24 (0.18,0.30) | 0.22 (0.17,0.28) | -0.02 (-0.06,0.01) | -0.01 (-0.03,0.01) | | 0.02 (-0.01,0.05) | 0.19 (0.13,0.24) | 0.03 (-0.02,0.08) | 0.04 (0.01,0.07) |
| Diseases of the blood and blood-forming organs and certain disorders involving the immune mechanism | 0.00 (-0.01,0.01) | 0.00 (0.00,0.01) | 0.00 (0.00,0.00) | 0.00 (-0.01,0.00) | 0.00 (0.00,0.00) | 0.00 (-0.01,0.01) | | 0.00 (0.00,0.01) | 0.00 (0.00,0.01) | 0.00 (0.00,0.00) | 0.00 (0.00,0.00) |
| Endocrine, nutritional, and metabolic diseases | 0.00 (-0.01,0.01) | 0.02 (0.00,0.03) | 0.08 (0.06,0.10) | -0.03 (-0.05,-0.02) | -0.02 (-0.03,-0.01) | 0.00 (-0.01,0.02) | | 0.00 (-0.01,0.01) | 0.02 (0.00,0.04) | -0.01 (-0.02,0.01) | 0.00 (-0.01,0.02) |
| Mental and behavioral disorders | 0.00 (0.00,0.00) | 0.02 (0.01,0.03) | 0.03 (0.02,0.05) | 0.00 (-0.01,0.01) | 0.00 (-0.01,0.01) | 0.00 (0.00,0.00) | | 0.00 (0.00,0.01) | 0.02 (0.01,0.04) | 0.00 (-0.01,0.00) | 0.01 (0.00,0.02) |
| Diseases of the nervous system | 0.01 (-0.01,0.03) | 0.04 (0.02,0.06) | 0.02 (0.01,0.04) | -0.01 (-0.02,0.00) | -0.01 (-0.02,0.01) | 0.01 (-0.01,0.04) | | 0.01 (0.00,0.03) | 0.02 (0.00,0.03) | -0.02 (-0.04,-0.01) | -0.04 (-0.05,-0.02) |
| Diseases of the circulatory system | 0.00 (-0.01,0.02) | 0.02 (0.00,0.04) | 0.18 (0.14,0.23) | -0.02 (-0.06,0.02) | 0.00 (-0.03,0.04) | 0.00 (-0.01,0.02) | | 0.02 (-0.01,0.05) | 0.08 (0.04,0.12) | 0.00 (-0.03,0.03) | 0.06 (0.02,0.09) |
| Diseases of the respiratory system | 0.00 (-0.01,0.02) | 0.01 (0.00,0.03) | 0.06 (0.04,0.08) | 0.09 (0.06,0.11) | 0.05 (0.02,0.08) | 0.01 (-0.01,0.03) | | 0.02 (0.00,0.04) | 0.08 (0.06,0.10) | 0.14 (0.12,0.18) | 0.13 (0.10,0.17) |
| Diseases of the digestive system | 0.00 (-0.01,0.00) | 0.04 (0.02,0.05) | 0.22 (0.19,0.26) | 0.03 (0.01,0.05) | 0.02 (0.00,0.03) | 0.00 (0.00,0.00) | | 0.01 (0.00,0.03) | 0.12 (0.09,0.15) | 0.03 (0.02,0.05) | 0.01 (0.00,0.03) |
| Diseases of the musculoskeletal system and connective tissue | 0.00 (0.00,0.00) | 0.00 (0.00,0.01) | 0.01 (0.00,0.01) | 0.01 (0.00,0.01) | 0.01 (0.00,0.02) | 0.00 (0.00,0.00) | | 0.00 (0.00,0.00) | 0.01 (0.00,0.01) | 0.01 (0.00,0.01) | 0.00 (-0.01,0.00) |
| Diseases of the genitourinary system | 0.01 (0.00,0.02) | 0.01 (0.00,0.01) | 0.01 (0.00,0.03) | -0.01 (-0.02,0.00) | -0.01 (-0.02,0.00) | 0.00 (0.00,0.00) | | 0.00 (0.00,0.01) | 0.01 (0.00,0.02) | 0.01 (0.00,0.02) | -0.01 (-0.02,0.01) |
| Symptoms, signs, and abnormal clinical and laboratory findings | 0.03 (0.00,0.06) | 0.03 (0.01,0.05) | 0.02 (0.00,0.05) | 0.06 (0.04,0.09) | 0.10 (0.07,0.14) | -0.01 (-0.04,0.03) | | 0.03 (0.01,0.05) | 0.13 (0.10,0.16) | 0.04 (0.02,0.06) | -0.03 (-0.05,0.00) |
| External causes of mortality | 0.06 (0.02,0.10) | 0.46 (0.38,0.54) | 0.50 (0.43,0.56) | 0.20 (0.17,0.23) | 0.03 (0.02,0.05) | 0.04 (0.00,0.09) | | 0.17 (0.09,0.24) | 0.26 (0.20,0.31) | 0.13 (0.11,0.16) | 0.05 (0.04,0.07) |
| Others | 0.02 (-0.04,0.09) | 0.00 (0.00,0.00) | 0.00 (0.00,0.01) | 0.00 (0.00,0.00) | 0.00 (-0.01,0.00) | 0.00 (-0.07,0.07) | | 0.00 (-0.01,0.00) | 0.00 (0.00,0.00) | 0.00 (0.00,0.01) | 0.00 (0.00,0.00) |
| **Women** |  |  |  |  |  |  | |  |  |  |  |
| Certain infectious and parasitic diseases | 0.00 (-0.01,0.01) | 0.01 (0.00,0.02) | 0.01 (0.00,0.03) | 0.00 (-0.01,0.02) | -0.01 (-0.02,0.01) | 0.00 (-0.01,0.02) | | 0.00 (-0.01,0.00) | 0.02 (0.01,0.04) | 0.02 (0.01,0.04) | 0.04 (0.02,0.06) |
| Neoplasms | -0.01 (-0.03,0.02) | 0.06 (0.02,0.10) | 0.04 (-0.02,0.10) | -0.11 (-0.15,-0.07) | -0.04 (-0.08,-0.01) | 0.01 (-0.01,0.03) | | 0.03 (0.00,0.07) | 0.08 (0.02,0.13) | -0.03 (-0.07,0.01) | -0.08 (-0.11,-0.05) |
| Diseases of the blood and blood-forming organs and certain disorders involving the immune mechanism | 0.00 (0.00,0.01) | 0.00 (0.00,0.01) | 0.00 (0.00,0.01) | 0.00 (0.00,0.00) | 0.00 (-0.01,0.00) | 0.00 (-0.01,0.00) | | 0.00 (0.00,0.00) | 0.01 (0.00,0.01) | 0.00 (0.00,0.00) | 0.00 (0.00,0.01) |
| Endocrine, nutritional, and metabolic diseases | 0.00 (0.00,0.00) | 0.01 (0.00,0.02) | 0.02 (0.01,0.04) | -0.04 (-0.06,-0.01) | -0.04 (-0.06,-0.03) | 0.01 (0.00,0.02) | | 0.00 (-0.01,0.01) | 0.01 (0.00,0.02) | 0.01 (0.00,0.03) | -0.03 (-0.05,-0.02) |
| Mental and behavioral disorders | 0.00 (0.00,0.01) | 0.01 (0.00,0.02) | 0.01 (0.00,0.02) | 0.00 (0.00,0.01) | 0.00 (-0.02,0.02) | 0.00 (0.00,0.00) | | 0.00 (0.00,0.01) | 0.00 (0.00,0.01) | 0.01 (0.00,0.01) | -0.03 (-0.04,-0.02) |
| Diseases of the nervous system | 0.00 (-0.02,0.03) | 0.02 (0.01,0.04) | 0.02 (0.00,0.03) | -0.01 (-0.02,0.00) | 0.00 (-0.02,0.02) | 0.01 (-0.01,0.03) | | 0.01 (-0.01,0.02) | 0.00 (-0.01,0.01) | -0.01 (-0.03,0.00) | -0.07 (-0.09,-0.04) |
| Diseases of the circulatory system | 0.00 (-0.02,0.02) | 0.01 (-0.01,0.03) | 0.10 (0.06,0.13) | -0.01 (-0.05,0.04) | -0.03 (-0.08,0.02) | 0.00 (-0.01,0.01) | | 0.01 (-0.01,0.03) | 0.06 (0.03,0.09) | 0.07 (0.04,0.11) | 0.03 (-0.02,0.07) |
| Diseases of the respiratory system | 0.01 (-0.01,0.03) | 0.01 (0.00,0.03) | 0.01 (0.00,0.02) | 0.01 (0.00,0.03) | 0.03 (0.00,0.06) | -0.01 (-0.01,0.00) | | 0.00 (-0.01,0.01) | 0.03 (0.01,0.04) | 0.04 (0.02,0.06) | 0.08 (0.05,0.12) |
| Diseases of the digestive system | 0.01 (0.00,0.03) | 0.02 (0.00,0.04) | 0.03 (0.01,0.05) | 0.00 (-0.01,0.02) | 0.01 (-0.01,0.02) | 0.00 (0.00,0.00) | | 0.01 (-0.01,0.02) | 0.05 (0.02,0.07) | 0.01 (-0.01,0.02) | 0.01 (-0.01,0.02) |
| Diseases of the musculoskeletal system and connective tissue | 0.00 (0.00,0.00) | 0.00 (-0.01,0.00) | 0.00 (-0.01,0.01) | 0.01 (0.00,0.02) | 0.03 (0.02,0.05) | 0.00 (0.00,0.00) | | 0.00 (-0.01,0.01) | 0.01 (0.00,0.02) | 0.00 (0.00,0.01) | 0.00 (-0.01,0.00) |
| Diseases of the genitourinary system | 0.01 (0.00,0.02) | 0.01 (0.00,0.02) | 0.01 (0.00,0.03) | -0.03 (-0.04,-0.01) | -0.02 (-0.04,-0.01) | 0.00 (0.00,0.00) | | 0.00 (0.00,0.01) | 0.01 (0.00,0.02) | 0.01 (-0.01,0.02) | 0.00 (-0.02,0.01) |
| Pregnancy, childbirth, and the puerperium | 0.00 (0.00,0.00) | -0.01 (-0.01,0.00) | 0.00 (0.00,0.00) | 0.00 (0.00,0.00) | 0.00 (0.00,0.00) | 0.00 (0.00,0.00) | | 0.00 (0.00,0.02) | 0.00 (0.00,0.00) | 0.00 (0.00,0.00) | 0.00 (0.00,0.00) |
| Symptoms, signs, and abnormal clinical and laboratory findings | 0.03 (-0.01,0.06) | 0.02 (0.00,0.04) | 0.01 (0.00,0.03) | 0.04 (0.02,0.06) | 0.10 (0.04,0.14) | 0.01 (-0.03,0.05) | | 0.00 (-0.02,0.01) | 0.03 (0.01,0.05) | 0.03 (0.01,0.05) | -0.13 (-0.16,-0.09) |
| External causes of mortality | 0.01 (-0.02,0.06) | 0.11 (0.04,0.18) | 0.15 (0.11,0.20) | 0.07 (0.05,0.09) | 0.03 (0.01,0.05) | 0.02 (-0.02,0.06) | | -0.04 (-0.09,0.02) | 0.06 (0.02,0.09) | 0.04 (0.02,0.06) | 0.01 (0.00,0.03) |
| Others | 0.06 (-0.01,0.14) | 0.00 (0.00,0.01) | 0.00 (0.00,0.01) | 0.00 (-0.01,0.00) | 0.00 (0.00,0.01) | 0.06 (-0.02,0.14) | | 0.00 (0.00,0.01) | 0.01 (0.00,0.01) | 0.00 (0.00,0.01) | 0.01 (0.00,0.02) |

The numbers in parentheses indicate the boundary values of the 95% uncertainty interval.


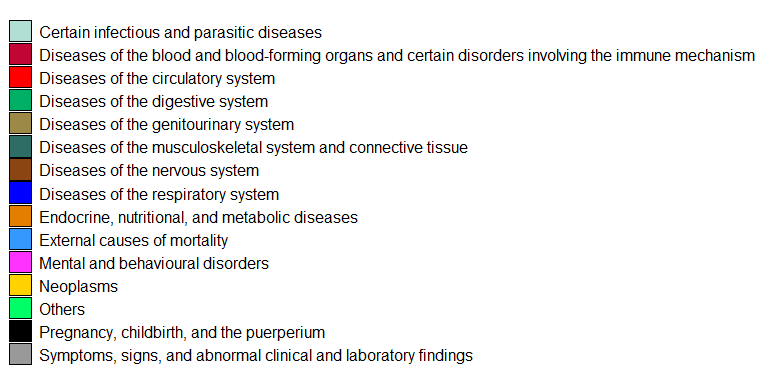

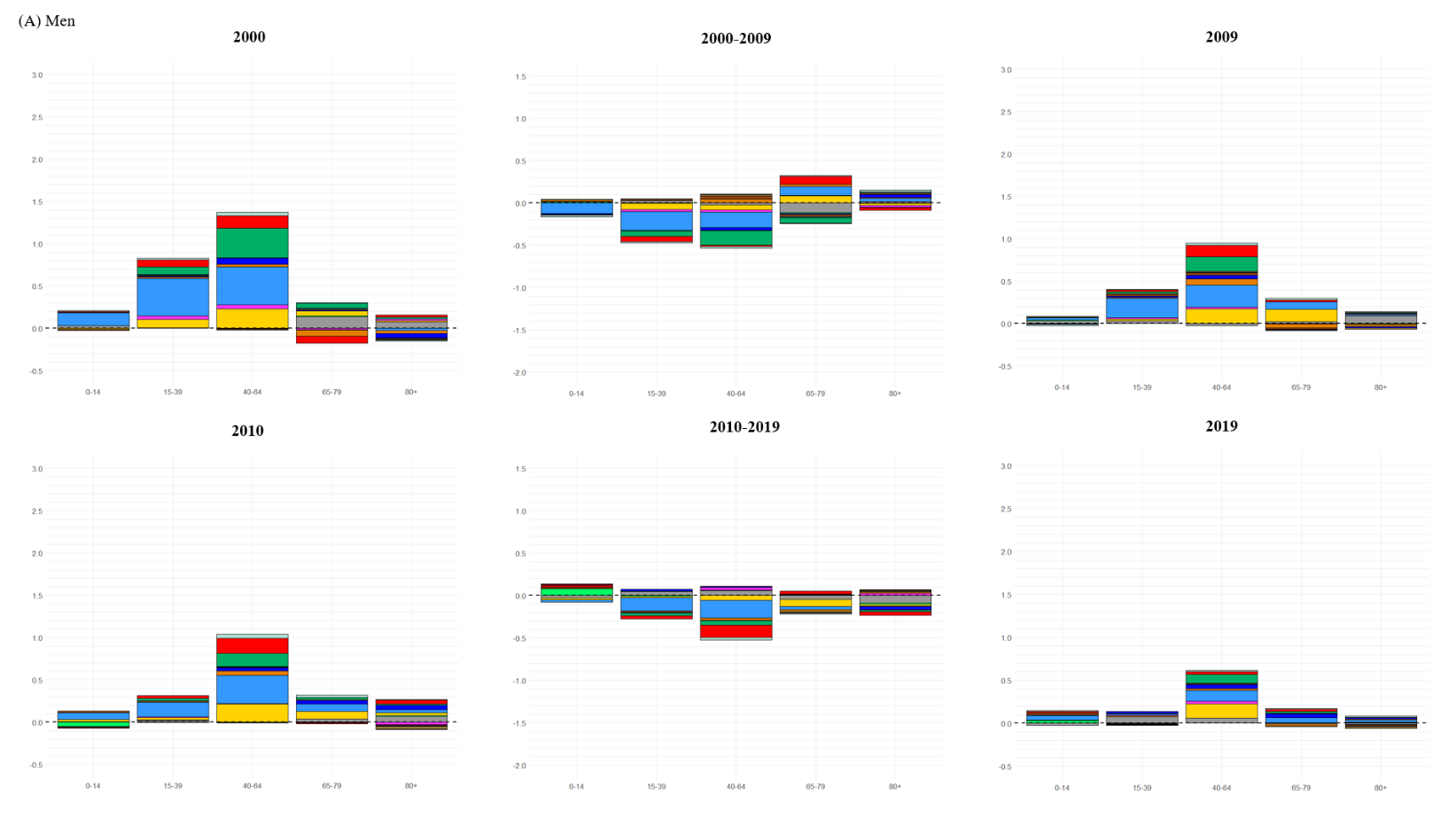


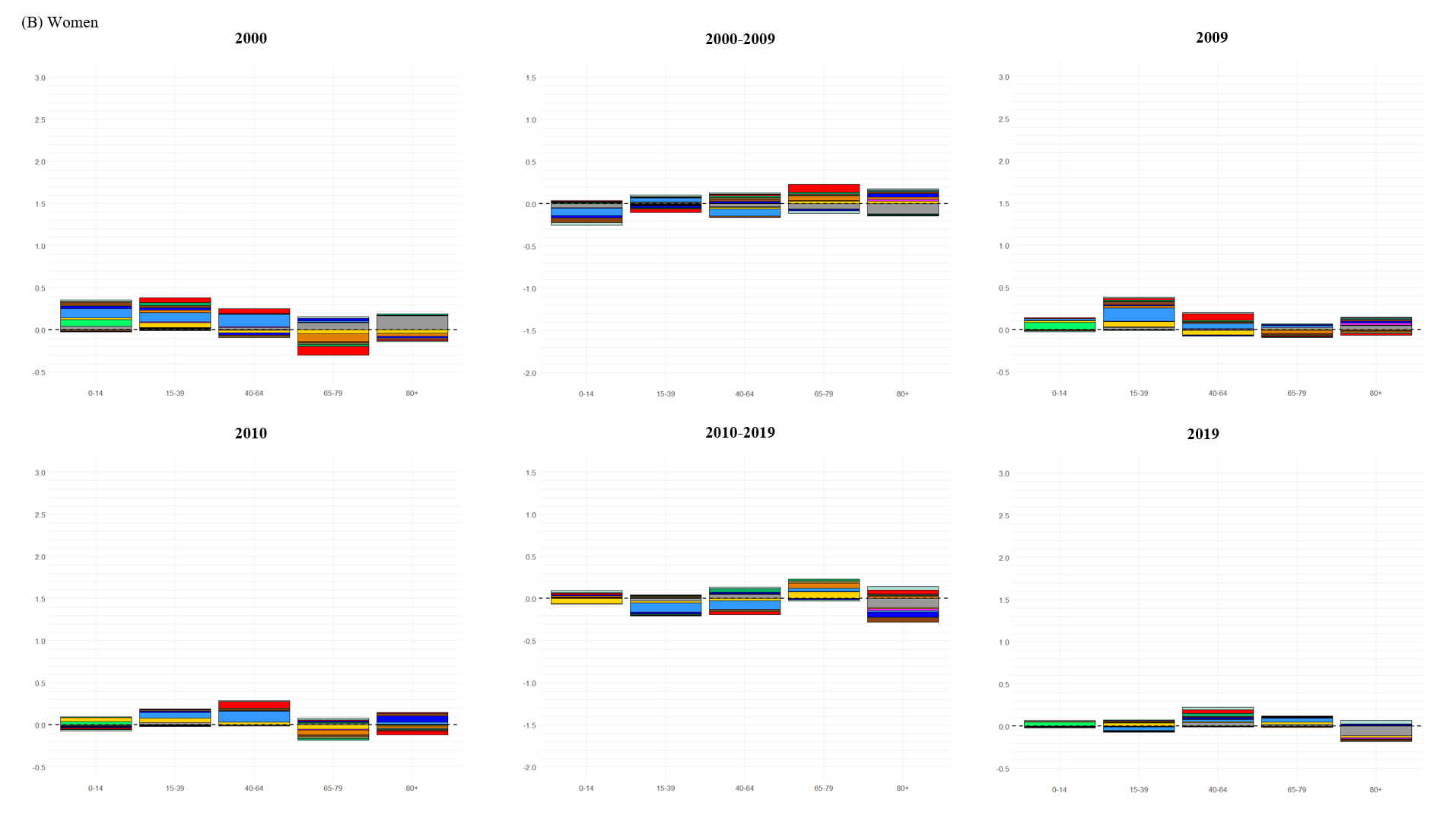


Supplementary Figure S2. Age- and cause-specific contribution to the life expectancy (LE) gap between small-to-middle urban areas (SMUAs) and rural areas (RAs) in 2000−2009 and 2010−2019: Findings from the Korean Statistical Information Services, Men and women.

A positive direction indicates a higher LE in the SMUAs and a negative direction indicates a higher LE in the RAs. The middle two panels represent the differences in LE changes between 2000−2009 and 2010−2019. The positive direction also indicated higher LE increases in the SMUAs, whereas the negative direction indicated higher LE increases in the RAs.


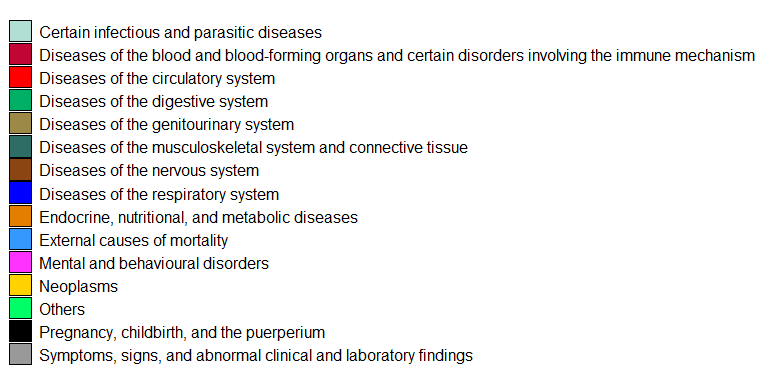

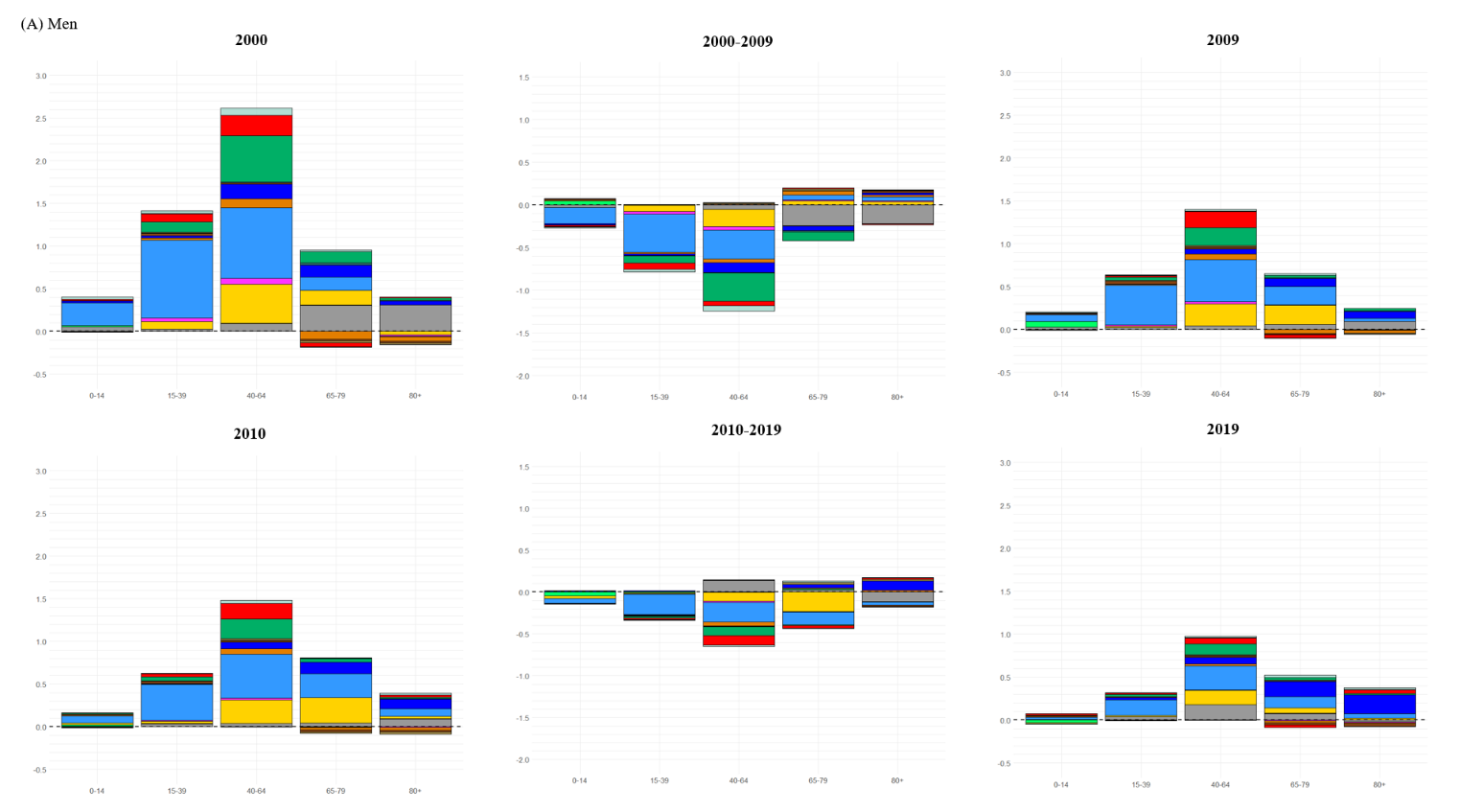


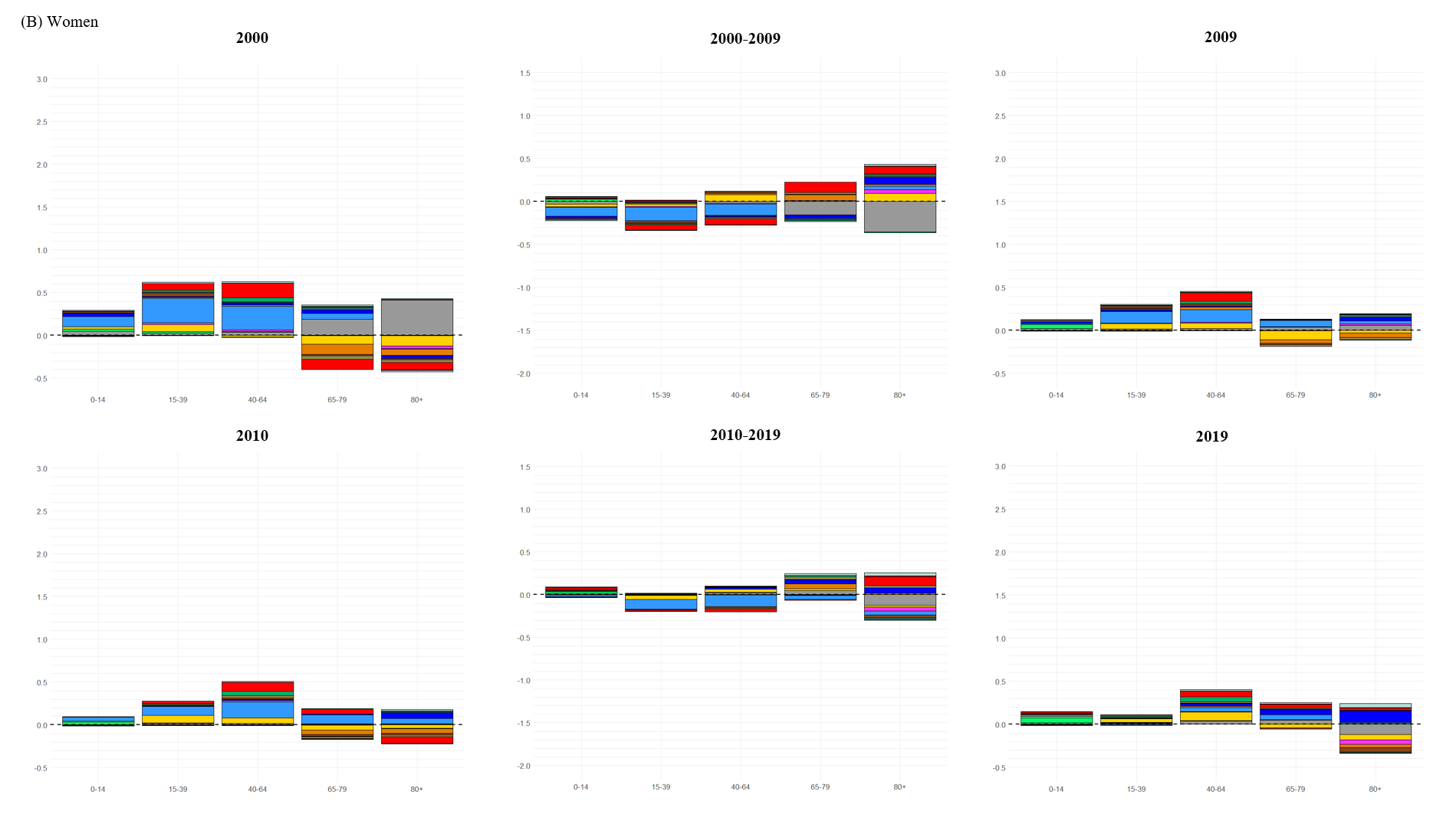


Supplementary Figure S3. Age- and cause-specific contribution to the life expectancy (LE) gap between large urban areas (LUAs) and rural areas (RAs) reclassified according to population density in 2000−2009 and 2010−2019: Findings from the Korean Statistical Information Services, Men and women.

A positive direction indicates a higher LE in the LUAs and a negative direction indicates a higher LE in the RAs. The middle two panels represent the differences in LE changes between 2000−2009 and 2010−2019. The positive direction also indicates higher LE increases in the LUAs, and the negative direction indicates higher LE increases in the RAs.
